# Mental health in UK Biobank – development, implementation and results from an online questionnaire completed by 157 366 participants: a reanalysis

**DOI:** 10.1192/bjo.2019.100

**Published:** 2020-02-06

**Authors:** Katrina A. S. Davis, Jonathan R. I. Coleman, Mark Adams, Naomi Allen, Gerome Breen, Breda Cullen, Chris Dickens, Elaine Fox, Nick Graham, Jo Holliday, Louise M. Howard, Ann John, William Lee, Rose McCabe, Andrew McIntosh, Robert Pearsall, Daniel J. Smith, Cathie Sudlow, Joey Ward, Stan Zammit, Matthew Hotopf

**Affiliations:** Researcher, Institute of Psychiatry, Psychology and Neuroscience, King's College London; and South London and Maudsley NHS Foundation Trust, NIHR Biomedical Research Centre, UK; Lecturer in Statistical Genetics, Institute of Psychiatry, Psychology and Neuroscience, King's College London; and South London and Maudsley NHS Foundation Trust, NIHR Biomedical Research Centre, UK; Data Scientist, Division of Psychiatry, University of Edinburgh, UK; Professor, University of Oxford; and Chief Scientist, UK Biobank, Nuffield Department of Population Health, University of Oxford Big Data Institute, UK; Professor of Psychiatric Genetics, Institute of Psychiatry, Psychology and Neuroscience, King's College London; and South London and Maudsley NHS Foundation Trust, NIHR Biomedical Research Centre, UK; Senior Lecturer, Institute of Health and Wellbeing, University of Glasgow, UK; Professor of Psychological Medicine, Institute of Health Research, University of Exeter Medical School, University of Exeter, UK; Professor of Psychology and Affective Neuroscience, Department of Experimental Psychology, University of Oxford, UK; Clinical Lecturer in General Psychiatry, Institute of Health and Wellbeing, University of Glasgow, UK; Senior Research Facilitator, University of Oxford; and UK Biobank: UK Biobank, Nuffield Department of Population Health, University of Oxford Big Data Institute, UK; NIHR Research Professor in Women's Mental Health and NIHR Senior Investigator, Section of Women's Mental Health, Institute of Psychiatry, Psychology and Neuroscience, King's College London, UK; Professor of Public Health and Psychiatry and Consultant Public Health Medicine, Population Data Science, Farr Institute of Health Informatics Research, Swansea University Medical School, Swansea University; and Public Health Wales NHS Trust, UK; Consultant Liaison Psychiatrist and Honorary Clinical Senior Lecturer, Devon Partnership NHS Trust; and University of Exeter Medical School, University of Exeter, UK; Professor of Clinical Communication, School of Health Sciences, City, University of London, UK; Professor of Biological Psychiatry, Division of Psychiatry, University of Edinburgh, UK; Consultant Psychiatrist and Honorary Clinical Senior Lecturer in Psychiatry, Institute of Health and Wellbeing, University of Glasgow, UK; Lecturer in Psychiatry, Institute of Health and Wellbeing, University of Glasgow, UK; Director of the British Heart Foundation Data Science Centre, BHF Data Science Centre; Former Chief Scientist, UK Biobank; and Chair of Neurology and Clinical Epidemiology, Centre for Medical Informatics, Usher Institute of Population Health Sciences and Informatics, University of Edinburgh, UK; Researcher, Institute of Health and Wellbeing, University of Glasgow, UK; Professor of Psychiatric Epidemiology, Centre for Academic Mental Health, University of Bristol; and Institute of Psychological Medicine and Clinical Neurosciences, University of Cardiff, Cardiff University School of Medicine, UK; Director, National Institute of Health Research Biomedical Research Centre at the Maudsley; Institute of Psychiatry, Psychology and Neuroscience, King's College London; and South London and Maudsley NHS Foundation Trust, NIHR Biomedical Research Centre, UK

**Keywords:** Mental health, UK Biobank, cohort study, depressive disorders, alcohol disorders

## Abstract

**Background:**

UK Biobank is a well-characterised cohort of over 500 000 participants including genetics, environmental data and imaging. An online mental health questionnaire was designed for UK Biobank participants to expand its potential.

**Aims:**

Describe the development, implementation and results of this questionnaire.

**Method:**

An expert working group designed the questionnaire, using established measures where possible, and consulting a patient group. Operational criteria were agreed for defining likely disorder and risk states, including lifetime depression, mania/hypomania, generalised anxiety disorder, unusual experiences and self-harm, and current post-traumatic stress and hazardous/harmful alcohol use.

**Results:**

A total of 157 366 completed online questionnaires were available by August 2017. Participants were aged 45–82 (53% were ≥65 years) and 57% women. Comparison of self-reported diagnosed mental disorder with a contemporary study shows a similar prevalence, despite respondents being of higher average socioeconomic status. Lifetime depression was a common finding, with 24% (37 434) of participants meeting criteria and current hazardous/harmful alcohol use criteria were met by 21% (32 602), whereas other criteria were met by less than 8% of the participants. There was extensive comorbidity among the syndromes. Mental disorders were associated with a high neuroticism score, adverse life events and long-term illness; addiction and bipolar affective disorder in particular were associated with measures of deprivation.

**Conclusions:**

The UK Biobank questionnaire represents a very large mental health survey in itself, and the results presented here show high face validity, although caution is needed because of selection bias. Built into UK Biobank, these data intersect with other health data to offer unparalleled potential for crosscutting biomedical research involving mental health.

## UK Biobank

UK Biobank is a very large, population-based cohort study established to identify the determinants of common life-threatening and disabling conditions.^1^ Most of these conditions, such as heart disease, stroke and mental disorders, are multifactorial, involving multiple genes of small effect, and complex relationships with environmental exposures. This means large samples are required to study associations between these exposures and disease, and to identify targets for treatment and prevention.^2^ The utility of traditional epidemiological study designs are often limited by their focus on single disorders or exposures and relatively modest sample sizes.^3^ UK Biobank is an open-access resource providing detailed characterisation of over half a million people aged 40–69 years at recruitment, with proposed long-term follow-up. Recruitment was completed in 2010, along with consent for future contact and linkage to routinely collected health-related data, such as those produced by the National Health Service (NHS). Baseline measures were extensive, from family history to sensory acuity (a searchable breakdown is available at www.ukbiobank.ac.uk), and the resource continues to grow. In 2017 genotyping of the whole cohort was complete, a range of blood biomarkers were released in 2019, and multimodal imaging is underway for 100 000 participants.^4^ Locality environmental factors, such as air pollution, are also available. The design of UK Biobank offers the opportunity to examine a wide range of risk factors and outcomes in a sample that has the size to provide the power to detect small effects, making UK Biobank a highly efficient resource for observational epidemiology.

The impact of mental disorders on disability and quality of life is considerable,^5^ accounting for the equivalent of over 1.2 million person-years lost to disability from mental and substance-use disorders in England alone in 2013.^6^ The detrimental impact of mental disorders both on physical disease onset and outcomes^7–9^ is particularly notable for this project. The UK Biobank baseline data collection of mental health, consisted of several questions about mood and a neuroticism scale, expanded for the last 172 729 recruited participants with questions to allow provisional categorisation of mood disorder;^10^ however, there was considerable scope for further characterisation of mental disorders among participants. The availability of mental health phenotypes in conjunction with the wealth of other data in the UK Biobank offers considerable opportunities to study aetiological and prognostic factors, particularly the interplay between factors that have usually been in separate research domains.^4^

## Outcome ascertainment

Characterising mental disorders in a cohort such as UK Biobank poses challenges. First, most mental disorders manifest before age 30 years and have fluctuating courses,^11^ so a ‘snapshot’ of disorder status at one point in time, as identified by most screening tools, is likely to be less useful than a ‘lifetime’ history – although ‘lifetime’ instruments suffer more from measurement error such as recall bias.^12,13^ Second, traditional diagnostic approaches to mental disorders, relying upon clinician assessment at interview, would be prohibitively expensive in a cohort of this size. Third, using self-report of diagnosis or data from record linkages relies upon recognition of illness and reflects healthcare usage patterns, whereas many people with mental disorders never seek or receive treatment.^11,14^ In response to these challenges, we developed a dual approach: secondary care record linkage for identification of more severe illnesses such as schizophrenia^15^ and self-report of symptoms of common mental disorder, which might not have come to clinical attention. As part of our mental health phenotyping programme we therefore developed an online mental health questionnaire (MHQ) for participants to complete regarding lifetime symptoms of mental disorders. The MHQ aimed to exploit the efficiency of ‘e-surveys’^16^ and provide the detail needed to identify mental health disorders without the need for a clinical assessment.

## Aims

The present paper aims to describe the development, implementation and results of the MHQ. We provide descriptive data on the numbers of UK Biobank participants meeting diagnostic criteria for specific disorders and on the frequency of exposure to risk factors. We also evaluate the likely representativeness of respondents by comparing respondent sociodemographic characteristics to that of the UK population using census data and comparing self-reported mental disorder diagnosis with the Health Survey for England (HSE) data.^17^ This will assist researchers considering or undertaking epidemiological research to evaluate the potential strengths and weaknesses of using UK Biobank data to look at mental health.

## Method

### Questionnaire development

A mental health research reference group formed of approximately 50 individuals (see supplementary Appendix 1 available at https://doi.org/10.1192/bjo.2019.100) participated in discussions about a strategy for mental health phenotyping in UK Biobank, including a workshop in January 2015. From this, a smaller steering group was established and led the development of the MHQ. The group recommended that the MHQ should concentrate on depression, as it was likely to represent the greatest burden in the cohort, with some questions about other common disorders, including anxiety, alcohol misuse and addiction, plus risk factors for mental disorders not captured at participants' baseline assessment.

The intention was to create a composite questionnaire out of previously existing and validated measures, taking into account participant acceptability (time, ease of use and ensuring questions were unlikely to offend), scope for collaborations with international studies (for example the Psychiatric Genomics Consortium) through making results comparable, and the need to balance depth and breadth of phenotyping. The base of the questionnaire was the measurement of lifetime depressive disorder using the Composite International Diagnostic Interview Short Form (CIDI-SF),^18^ modified to provide lifetime history, as used to identify cases and controls for some existing studies in the Psychiatric Genomics Consortium.^19^ The CIDI-SF uses a branching structure with screening questions and skip rules to limit detailed questions to the relevant areas for each participant. Other measures were then added to this, as summarised in supplementary Table SM1. Where the group were unable to find existing measures that fulfilled these criteria, questions were written or adapted, as indicated in supplementary Table SM1. These sections have not been externally validated, but the questions along with the full questionnaire can be seen on the UK Biobank website (http://biobank.ctsu.ox.ac.uk/crystal/refer.cgi?id=22), for researchers to evaluate.

### Testing and ethical approval

The use of branching questions in the MHQ means that those with established and multiple mental disorders have a longer, more detailed, questionnaire. To improve acceptability in this group, we worked with a patient advisory group at the National Institute of Health Research (NIHR) Biomedical Research Centre at the South London and Maudsley NHS Foundation Trust in designing the questionnaire and invitation.^20^ We then piloted the questionnaire for functionality (for example ease of completion) among an online cohort of 14 836 volunteers aged over 50 and living in the UK, who completed the questionnaire as part of signing up to take part in the Platform for Research Online to investigate Genetics and Cognition in Ageing (PROTECT).^21^ Of those who started the questionnaire 98.8% completed it, taking a median time of 15 min. Some PROTECT participants commented that they wanted the opportunity to explain why they felt they had experienced symptoms of depression. In response to this, we added a question to the depression section on loss or bereavement, and a free-text box – neither were designed to change diagnostic algorithms, but may add to future analyses.

The questionnaire was approved as a substantial amendment to UK Biobank approval from the North West - Haydock Research Ethics Committee**,** 11/NW/0382. Participation in the UK Biobank is voluntary, and participants are free to withdraw at any time. Informed written consent was obtained by participants at baseline. Online questionnaires such as the MHQ are voluntary.

### Administration to UK Biobank participants

We incorporated the final MHQ into the UK Biobank web-questionnaire platform and presented it to participants as an online questionnaire entitled ‘thoughts and feelings’. To participants who had agreed to email contact (339 092/503 328 participants, 67%) we sent a hyperlink to their personalised questionnaire. The invitation explained the importance of collecting further information about mental health and emphasised that UK Biobank was unable to respond to concerns raised by the participant in the questionnaire, instead directing them to several sources of potential support. Participants could skip questions they preferred not to answer, and they could save answers to return to the questionnaire later. We sent reminder emails at 2 weeks and 4 months to those who had not started or had partially completed the questionnaire. The MHQ will continue to be available on the participant area of the UK Biobank website, and since 2017 the annual postal newsletter contains an invitation to log on to the participant area and complete questionnaires, which will reach those for whom no email contact was possible. Data from the MHQ will therefore continue to accrue. The current numbers and aggregate data can be accessed from the public data showcase (http://biobank.ctsu.ox.ac.uk/crystal/label.cgi?id=136). More detail on the rollout and associated communications can be found on the UK Biobank website (http://biobank.ctsu.ox.ac.uk/crystal/refer.cgi?id=22).

### Defining outcomes from the MHQ

Some suggested case definitions for the evaluation of the responses on the MHQ are detailed in supplementary Appendix 2. They arose either from the instruments used in the MHQ or by consensus criteria agreed by the working committee who wrote the MHQ. Diagnostic criteria were evaluated for depression (major depressive disorder), hypomania or mania, generalised anxiety disorder (GAD), hazardous/harmful alcohol use (alcohol use disorder) and post-traumatic stress disorder (PTSD). Addiction to substances and/or behaviour was defined based on self-report alone. Unusual experiences (describing potential symptoms of psychosis) and self-harm were also defined as phenomena that are important for phenotyping, but are not specific to any disorder. We combined outcomes to divide the cohort into five mood disorder groups, as shown in supplementary Fig. MD1.

Fulfilling the diagnostic criteria based on a self-report questionnaire does not allow us to rule out other psychiatric disorders, psychological or situational factors that might better explain the symptoms, which may have been elicited had there been a clinical evaluation. Therefore, we would regard any case classification arising from the MHQ as ‘likely’, rather than a confirmed psychiatric disorder. The issue becomes particularly problematic for disorders that are less common in the population, such as bipolar affective disorder, where literature shows that using questionnaires to screen the population may overestimate prevalence.^22^ Therefore, although we report the presence of hypomania/mania symptoms for the whole population, we only make the likely diagnosis of bipolar affective disorder in people with a history of depression, a subpopulation where the prevalence of bipolar affective disorder is higher, and therefore screening questionnaires have better positive predictive values.^23^

### Analysis and data sharing

Data were supplied by UK Biobank on 8 August 2017 under application number 16577. This data is open-access subject to the usual access procedures (www.ukbiobank.ac.uk).

Formal operational criteria (supplementary Appendix 2) were written by K.A.S.D. based on consensus within the consortium (see Defining cases from the MHQ, above), with checks by J.R.I.C., G.B. and M.H. (whole) and C.D., N.G., W.L. and D.J.S. (mood disorders section). R code for analysis was developed by J.R.I.C., with the code posted for comments during development, trialled on pilot data and checked by K.A.S.D. and G.B. Portions of the data were analysed independently in parallel by other groups and subsequently compared (for example for mood disorders, by N.G./B.C.). The R code is freely available from Mendeley Data for the purpose of reproducing these analyses or developing further analyses (https://data.mendeley.com/datasets/kv677c2th4/3). We used R version 3.4.0–3.5.1 and MS Excel for analyses. We report numbers and proportions within the sample and do not attempt to give population prevalence estimates. Because of this, and the large sample size (the 95% CIs on all proportions have width of less than absolute 1%), CIs were thought not to add meaning, and so are not shown. A STrengthening the Reporting of OBservational studies in Epidemiology checklist is included in supplementary Appendix 3.

### Comparison data

In order to describe the differences in the sample of participants in UK Biobank to the general population of the UK, Fry *et al*^24^ compare UK Biobank data with census data, which we have replicated and extended. For health-related data, we have used the Health Survey for England (HSE), which is a face-to-face household survey carried out every year^25^ that in 2014 involved around 8000 adult participants designed to be representative of the England adult population (with weighting in cases where sampling could not achieve this). Some topics are ‘core’ and are surveyed every year, whereas others are ‘supplementary’. Mental health appeared in the 2014 survey as a supplementary topic.^17^

## Results

**Fig. 1:**
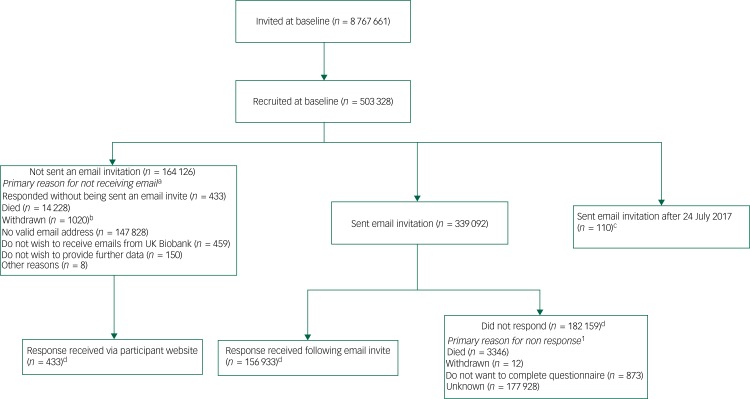
Flow chart of UK Biobank participants from invitation to completion of mental health questionnaire (MHQ). Invitations were based on National Health Service registration, age and location. Numbers correct for July 2017.

The setting, recruitment and methods of selection of participants in UK Biobank have been published elsewhere.^1,4,10^ For the MHQ study, 339 092 participants were sent an email invitation, and 157 366 (46% of those emailed) fully completed the questionnaire by July 2017 (available in August 2017) – which means that the MHQ had 31% coverage of the UK Biobank cohort. The coverage continues to grow as the questionnaire is still open for participants. Figure 1 shows the flow chart of UK Biobank participants who completed the MHQ. The median time for completion was 14 min, and 82% of respondents completed the questionnaire in under 25 min.

a. Participants could have multiple reasons for not being sent an email, or for not responding. For the purposes of this flow chart, we have identified the most important reason. b. Withdrawals include participants who requested no further use and no further access, plus requests for no further contact before 11 April 2017 (after which they are included in other categories). c. When data was accessed in August 2017, response data was available for invitations sent up to and including 24 July, whereas results were available for response up to 27 July, with 110 invitations being sent between these dates. d. The discrepancy between this figure and the 157 366 figure in the paper is because of responses between 24 and 27 July from those who previously had not responded or who had only just been emailed. The survey has remained open, and is accessible for participants without an email invitation, thus will continue to accrue data.

Supplementary Table SM2 shows participant characteristics for all UK Biobank participants and those who completed the MHQ compared with population-level data for UK residents in the same age range. The MHQ participants were aged 45–82 years, with 53% aged 65 or over, and 57% were female. They were different from the whole UK Biobank cohort and the general population by being better educated (for example 45% hold a degree *v.* 32% of all UK Biobank participants *v.* 23% in the census), of higher socioeconomic status according to job type, and healthier (28% report long-standing illness or disability *v.* 32% all UK Biobank participants *v.* 37% census), with lower rates of current smoking.

Table 1 shows that 34% of respondents reported they had received at least one psychiatric diagnosis from a professional at some time, and 12% had received two or more. The most commonly reported diagnosis was depression, followed by ‘anxiety or nerves’. Data are compared with the population prevalence estimates from HSE for this age group.^17^ The comparison shows that the pattern and prevalence of diagnosis are similar; for example, a depression diagnosis was self-reported by 21% of individuals in both samples, eating disorder by around 1% and bipolar-related disorders by around 0.5%. The definition in the MHQ differed from that in the HSE for anxiety (the MHQ definition was broader) and addiction (MHQ did not require professional diagnosis), and the higher overall prevalence in the UK Biobank MHQ compared with the HSE (34.3% *v*. 28.0%) may be a result of those wider definitions.

**Table 1:** Respondent reports of mental health diagnoses by a professional (self-reported without physician diagnosis for addiction) compared with diagnoses reported in the Health Survey for England (HSE) 2014^a^

	UK Biobank MHQ responses, age 45–82 years (*n* = 157 366)		HSE, age 45–84 years (*n* = 3272)	
	*n*	% in sample	*n*	Prevalence (95% CI)
All psychotic disorders	723	0.5	11	0.3 (0.2–0.6)
Schizophrenia	157	0.1	NR	–
Any other type of psychosis or psychotic illness	604	0.4	NR	–
Depression	33 424	21.2	679	20.8 (19.4–22.2)
Mania, hypomania, bipolar or manic-depression	837	0.5	13	0.4 (0.2–0.7)
Anxiety, nerves or generalised anxiety disorder^b^	22 036	14.0	170	5.2 (4.5–6.0)
Panic attacks	8704	5.5	262	8.0 (7.1–9.0)
Agoraphobia	599	0.4	NR	–
Social anxiety or social phobia	1962	1.2	NR	–
Any other phobia (for example disabling fear of heights or spiders)	2153	1.4	27	0.8 (0.6–1.2)
Obsessive–compulsive disorder	982	0.6	11	0.3 (0.2–0.6)
A personality disorder	385	0.2	13	0.4 (0.2–0.7)
All eating disorders	1851	1.2	26	0.8 (0.5–1.2)
Anorexia nervosa	891	0.6	NR	–
Bulimia nervosa	503	0.3	NR	–
Psychological overeating or binge-eating	707	0.4	NR	–
Autism, Asperger's or autistic spectrum disorder	223	0.1	NR	–
Attention-deficit or attention-deficit hyperactivity disorder	133	0.1	4	0.1 (0–0.3)
Any addiction or dependence	9386	6.0	NR	–
Alcohol or drug addiction^c^	5002	3.2	30	0.9 (0.6–1.3)
Physical alcohol dependence	946	0.6	NA	*–*
Summary				
None of above	103 346	65.7	2356	72.0 (70.4–73.5)
≥1 of above	54 020	34.3	916	28.0 (26.5–29.6)
≥2 of above	19 400	12.3	NR	–

MHQ, mental health questionnaire; NR, not reported.

a. UK Biobank participants were asked: ‘Have you been diagnosed with one or more of the following mental health problems by a professional, even if you don't have it currently? (tick all that apply): By professional we mean: any doctor, nurse or person with specialist training (such as a psychologist or therapist). Please include disorders even if you did not need treatment for them or if you did not agree with the diagnosis’. HSE participants were asked to identify all the mental health conditions they had experienced, then asked whether they had been told by a doctor, psychiatrist or professional that they had it.

b. HSE participants were asked about generalised anxiety disorder, and not about anxiety and nerves more generically.

c. UK Biobank participants asked: ‘Have you been addicted to or dependent on one or more things, including substances (not cigarettes/coffee) or behaviours (such as gambling)?’ HSE definition of addiction includes physician diagnosis.

Table 2 shows that 45% of participants met criteria for one or more operationally defined syndromes. Of the lifetime disorders, depression was most common (24% respondents participants), then GAD (7%) and hypomania/mania (2%); current hazardous/harmful alcohol use was met by 21% and current PTSD by 6%. Lifetime unusual experiences were reported by 5% of respondents and self-harm by 4%. Supplementary Table SM3 shows that women and men were approximately equally likely to have a history of one or more of the defined syndromes (women 44% *v.* men 46%), but differed as to which criteria were met: women were more likely to have a history of depression or anxiety disorder, whereas men were more likely to meet criteria for a current hazardous/harmful alcohol use (women 14% *v.* men 30%). Table 2 also shows the substantial comorbidity of defined syndromes. Notably, around three-quarters of participants who met criteria for lifetime anxiety disorder also met criteria for lifetime depression. Also, although individuals meeting criteria for PTSD had more than a twofold risk of all of the lifetime syndromes compared with average, those identified with hazardous/harmful alcohol use had little extra risk of lifetime syndromes.

**Table 2:** Comorbidity between operationally defined syndromes^a^

	Overall Prevalence, *n* (%) (*n* = 157 366)	Comorbidity, *n* (%)						
		Depression^b^	Hypomania/mania^c^	Anxiety disorder^d^	Unusual experiences^e^	Self-harm^f^	Haz/harm alcohol use^g^	PTSD^h^
Total	70 892 (45)	37 434 (24)	2396 (2)	11 111 (7)	7803 (5)	6872 (4)	32 602 (21)	10 064 (6)
Lifetime history								
Depression^b^	37 434 (24)	–	1550 (4)	8444 (23)	3649 (10)	4240 (11)	8156 (22)	6373 (17)
Hypomania/mania^c^	2396 (2)	1550 (65)	–	778 (32)	598 (25)	453 (19)	660 (28)	657 (27)
Anxiety disorder^d^	11 111 (7)	8444 (76)	778 (7)	–	1551 (14)	1704 (15)	2634 (24)	3274 (29)
Unusual experiences^e^	7803 (5)	3649 (47)	598 (8)	1551 (20)	–	1225 (16)	1784 (23)	1594 (20)
Self-harm^f^	6872 (4)	4240 (62)	453 (7)	1704 (25)	1225 (18)	–	1890 (28)	1719 (25)
Current								
Hazardous/harmful alcohol use^g^	32 602 (21)	8156 (25)	660 (2)	2634 (8)	1784 (5)	1890 (6)	–	2572 (8)
PTSD^h^	10 064 (6)	6373 (63)	657 (7)	3274 (33)	1594 (16)	1719 (17)	2572 (26)	–

PTSD, post-traumatic stress disorder.

a. Percentages refer to the proportion of participants with the row syndrome who also have column syndrome. See footnotes b–h, and supplementary Appendix 2 for ‘case’ definitions.

b. Criteria met for major depressive disorder on Composite International Diagnostic Interview Short Form (CIDI-SF) lifetime.

c. Criteria met for hypomania/mania lasting for at least 1 week.

d. Criteria met for generalised anxiety disorder on CIDI-SF lifetime.

e. Reported potential hallucination or delusion at any point in their life.

f. Reported self-harm at some point in their life, asked to report self-harm ‘whether or not you meant to end your life’.

g. Score above cut-off for alcohol use disorder (≥8) on Alcohol Use Disorder Identification Tool during the past year.

h. Criteria met for post-traumatic stress disorder (PTSD) on PTSD Checklist – Short version (PCL-S) in the past month.

In Table 3, people meeting criteria for the lifetime occurrence of at least one of depression, bipolar disorder, GAD, unusual experiences or self-reported addiction are seen to be more likely than those without to come from a younger age group, report adverse life events and have met criteria for loneliness or social isolation. They are more likely to have smoked cigarettes and/or used cannabis, and to have had a ‘longstanding illness’ at baseline (although the presence of a mental disorder may have been the illness to which the participants refer in some cases), but all groups were equally likely to be achieving recommended levels of physical activity. Markers of deprivation (area-level deprivation and rented housing) are raised in groups with a history of mental disorders, especially bipolar affective disorder and addictions.

**Table 3:** Selected personal characteristics, socioeconomic factors, risk factors and health behaviours by status for likely lifetime occurrence of operationally defined syndromes (people may be included in more than one category)

Characteristics		No ‘lifetime’ criteria met^a^ (*n* = 108 752)	Depression^b^ (*n* = 37 434)	Bipolar type 1^c^ (*n* = 931)	Anxiety disorder (GAD)^b^ (*n* = 11 111)	Unusual experiences^d^ (*n* = 7803)	Addiction^e^ (*n* = 9386)
*Personal characteristics*							
Age,^f^ *n* (%)	45–54	14 364 (13)	7145 (19)	228 (24)	2348 (21)	1485 (19)	2013 (21)
	55–64	33 307 (31)	14 809 (40)	417 (45)	4470 (40)	2904 (37)	3428 (37)
	65–74	51 705 (48)	13 739 (37)	261 (28)	3892 (35)	2960 (38)	3466 (37)
	≥75 (oldest is 82)	9376 (9)	1741 (5)	25 (3)	401 (4)	454 (6)	479 (5)
Gender, *n* (%)	Female	57 556 (53)	25 815 (69)	532 (57)	7404 (67)	4718 (60)	4556 (49)
Ethnicity, *n* (%)	White	105 072 (97)	36 297 (97)	892 (96)	10 749 (97)	7503 (96)	9037 (96)
Townsend Deprivation Score,^g^ *n* (%)	Most deprived (TDS ≥2)	11 783 (11)	5656 (15)	201 (22)	1856 (17)	1426 (18)	1941 (21)
Highest qualification, *n* (%)	Degree	48 700 (45)	16 939 (45)	425 (46)	5071 (46)	3646 (47)	4531 (48)
Housing tenure, *n* (%)	Rent^h^	4162 (4)	2906 (8)	155 (17)	1026 (9)	854 (11)	1109 (12)
*Known risk factors*							
Neuroticism score,^i^ mean (s.d.)		3.2 (2.8)	5.6 (3.3)	3.8 (3.1)	7.1 (3.3)	5.2 (3.5)	5.4 (3.5)
Adverse life experiences, *n* (%)	Childhood screen^j^	43 913 (40)	21 144 (56)	638 (69)	6931 (62)	4783 (61)	5800 (62)
	Adult screen^k^	50 226 (46)	23 893 (64)	685 (74)	7581 (68)	5284 (68)	6303 (67)
	Trauma exposure^l^	50 771 (47)	22 166 (59)	665 (71)	6877 (62)	5439 (70)	6278 (67)
Social connection, *n* (%)	Loneliness^i^^,^^m^	2976 (3)	2367 (6)	94 (10)	971 (9)	570 (7)	669 (7)
	Social isolation^i^	7793 (7)	3827 (10)	126 (14)	1173 (11)	931 (12)	1200 (13)
Illness	Longstanding illness, disability or infirmity^i^	26 341 (24)	13 363 (36)	503 (54)	4581 (41)	3242 (42)	3588 (38)
*Health behaviours*							
Smoking status,^i^ *n* (%)	Current	6235 (6)	3638 (10)	158 (17)	1194 (11)	837 (11)	1916 (20)
Cannabis use (lifetime), *n* (%)	Daily	868 (1)	914 (2)	63 (7)	346 (3)	258 (3)	867 (9)
Physical activity,^i^ *n* (%)	Moderate activity ≥ three times a week	39 677 (36)	13 988 (37)	345 (37)	4174 (38)	2846 (36)	3602 (38)

a. Criteria not met for depression, generalised anxiety disorder (GAD), unusual experiences or addiction.

b. Criteria met for disorder on Composite International Diagnostic Interview Short Form (CIDI-SF) lifetime.

c. Criteria met for both lifetime depression and lifetime mania.

d. Reported potential hallucination or delusion at any point in their life.

e. Positively endorsed: ‘Have you been addicted to or dependent on one or more things, including substances (not cigarettes/coffee) or behaviours (such as gambling)?’.

f. Age when mental health questionnaire completed, derived from date of birth.

g. Townsend Material Deprivation Score is based on postcode areas.

h. Includes rent social and rent private, excludes other categories of housing tenure.

i. From baseline assessment 2006–2010

j. Criteria met for possible abuse or neglect on Childhood Trauma Screener.

k. Criteria met for adverse situations as an adult: lack of confiding relationship, abusive relationships and money problems.

l. Reports one or more of six situations that are known to be triggers for trauma-related disorders.

m. There is some overlap between the adult screen and loneliness screen, which both ask about confiding relationships: adult screen includes lack of confiding relationship over the adult lifetime; loneliness includes lack of confiding relationship at the time of baseline assessment.

The supplementary material includes a section on mood disorder, showing the results of analyses of MHQ participants by likely disorder categories (supplementary Fig. MD1). Supplementary Table MD1 shows the features of these groups. The characteristics of people who meet diagnostic criteria for depression appear to be shared by those with subthreshold depressive symptoms. Supplementary Table MD2 shows comorbidity, and demonstrates a gradient effect in the presence of a non-depression syndrome rising from 23% in no depression (mainly hazardous/harmful alcohol use) to 50% in recurrent depression. Supplementary Table MD3 shows that people with a history of depression or bipolar affective disorder tend to have worse scores for current mental health.

## Discussion

### Main findings relating to data-collection methods

This paper has described the development, implementation and principal descriptive findings from the UK Biobank MHQ. The implementation of this questionnaire demonstrates that a web-based questionnaire is an acceptable means of collecting mental health information at low cost and large scale. Although the data-collection methods might force more limited data acquisition than conventional interview methods, with associated uncertainties in true diagnostic categorisation, we suggest that the survey achieved an acceptable trade-off between depth of phenotypic information and scale of sample size. This means that the UK Biobank MHQ sample can usefully fill a gap between clinical samples with detailed mental health disorder information but poor generalisability (for example, Clinical Records Interactive Search^26^) and larger cohorts with superficial identification of mental disorder (such as the baseline UK Biobank cohort or 23andMe^27^).

The MHQ achieved a participation rate of 31% of the original UK Biobank participants and 46% of those emailed. This response rate is substantially higher than previous UK Biobank questionnaires, largely owing to the attention paid to ensure the acceptability of the invitation and questionnaire and the efficient use of reminders.

### Main findings from the questionnaire

Those who completed the MHQ appear to be better educated and have higher socioeconomic status (job title, household income, home ownership and area-level deprivation) than those recruited into UK Biobank overall, and the UK population. Despite this, we found that rates of self-report diagnoses were similar to population estimates from the HSE. The patterns of association between disorders and demographics were also broadly as predicted by previous research, which adds to the face validity of the questionnaire. For example, depression and anxiety were more common in women, whereas addiction and alcohol misuse were more common in men, and all disorders were less common in respondents older than 65 years. The decrease in prevalence of lifetime disorder with increasing age has been previously noted in cross-sectional studies, although the causes and implications are not clearly understood.^28,29^ The high level of hazardous/harmful alcohol (using the Alcohol Use Disorder Identification Tool) is consistent with the Adult Psychiatric Morbidity Survey 2014, where they comment on increased numbers in older age groups since 2007.^30^

#### ‘Healthy volunteer’ selection bias

The ‘healthy volunteer’ selection bias within the UK Biobank has been previously explored,^24^ and further variables influencing participation in the MHQ can be predicted and have been found, such as an interest in mental health and good cognition.^31^ The impact of selection biases on disease prevalence are likely to be particularly strong for mental health disorders, where disorder status or symptoms may influence participation in research,^32,33^ and many risk factors for these disorders, including genetic risk, can be associated with non-participation.^34^ Therefore, the results of the MHQ should not be used to provide prevalence estimates. However, the pattern of the measured risk factors among the participants with mental disorders in the MHQ, including neuroticism, trauma, loneliness and housing tenure, was in accordance with established literature, supporting the use of the data to study the relationships between exposures and outcomes. Previous work on health surveys with selection bias because of non-participation, including UK Biobank, have indicated that they can be used to give estimates of association,^14,32,35^ although biased results may occur in some cases.^36,37^ For example, the relative under-participation of unskilled workers in the MHQ (around one-fifth of the proportion in the population) could mask an association with a variable that was related to unskilled work.

## Strengths and limitations

We developed a questionnaire through a consensus approach with clear aims of capturing enough data to characterise participants as having a lifetime history of depression and other phenotypes. Validated instruments were used where possible. The consortium working on the questionnaire included mental health researchers and members of the UK Biobank team working in collaboration to develop the optimum approach. The derived variables of likely categorical diagnoses will be added to the UK Biobank resource, facilitating those less familiar with mental health to use the results efficiently. The UK Biobank data, including that from the MHQ, is available to researchers and we have made the code used to derive the results in this paper freely available, allowing other researchers both to query our findings and build upon them for their own work.

The ‘healthy volunteer’ effect may limit applications of the data. The questionnaire was also heavily reliant on participant report, which may be affected by the stigma of reporting psychiatric symptoms, and tends to underestimate lifetime prevalence through forgetting or re-evaluating distant events.^14,28,38^ This caveat on ‘lifetime’ disorder is another reason this data is more suitable for association studies than prevalence estimates. Researchers considering the use of UK Biobank data will need to assess the likely impact of selection bias and recall bias on a case-by-case basis, as this will affect whether UK Biobank is suitable and the choice of mental health data within UK Biobank.^39^

As a result of restrictions of time and space, the questionnaire was limited in the topics it could cover. The focus of the questionnaire was on categorical diagnoses rather than dimensional traits, which will tend to confirm conventional ICD/DSM nosology of psychiatric disorder and may not suit some research.^40^ In particular, tools were chosen that are based on DSM-IV disorders, which reflects current practise (for example National Institute for Health and Care Excellence guidelines on depression and anxiety use DSM-IV definitions).^41,42^ Of the disorders with operational classification, all would generalise to DSM-5, except PTSD,^43^ and the concepts are valid for ICD-10 disorders, although the threshold of disorder may be different, for example depression is diagnosed with fewer symptoms in DSM than ICD.^41^ Developments in UK Biobank such as primary care linkage and proposed future questionnaires (such as quality of life, activities of daily living) will continue to enrich the picture of mental health in the cohort going forward.

## Implications

In conclusion, UK Biobank offers a unique opportunity to research common disorders in a well-characterised longitudinal cohort of UK adults. A detailed MHQ has now been completed by 157 366 participants, including self-report, operationally defined lifetime disorder status and detailed phenotype information on mood disorder. The proportion of participants with mental disorders and the patterns of participants experiencing symptoms and disorders was as expected despite a ‘healthy volunteer’ selection bias. Further work on mental health phenotyping for UK Biobank includes validation of Hospital Episode Statistics for mental health diagnoses,^15^ incorporation of general practice records, triangulation of health record and questionnaire data,^39^ and investigation of further putative phenotypes.^44^ Existing projects utilising UK Biobank mental health data can be seen in a searchable database of approved research (http://www.ukbiobank.ac.uk/approved-research/).

This study also demonstrates the substantial burden of mental health disorders, including potentially dangerous patterns of alcohol consumption. Given the known impact of mental health on physical health, mental health data and its associations should interest researchers from every biomedical speciality. This study suggests that UK Biobank could be a powerful tool for such studies, and as it is open to all *bona fide* health researchers for work in the public good, we hope this study will inspire both existing and new users of UK Biobank.

## Data Availability

Available from UK Biobank subject to standard procedures (www.ukbiobank.ac.uk). Code for replication available from Mendeley Data (http://doi.org/10.17632/kv677c2th4.3).

## Supplementary material

For supplementary material accompanying this paper visit http://dx.doi.org/10.1192/bjo.2019.100. click here to view supplementary material
