# Supplementary material for: Mental health in UK Biobank – development, implementation and results from an online questionnaire completed by 157 366 participants: a reanalysis
Source: BJPsych Open. 2020 Feb 6;6(2):e18. doi: 10.1192/bjo.2019.100 (PMC7176892; doi:10.1192/bjo.2019.100)
Supplement: Supplementary file 1 [file S2056472419001005sup001.docx]

Mental Health in UK Biobank Revised – development, implementation and results from an online questionnaire completed by 157,366 participants. Davis, Coleman, et al.

**Supplementary Material**

[Extra Tables and Figures](#extra)

- [Table SM1: The structure of the UK Biobank mental health questionnaire “thoughts and feelings”](#SM1)
- [Table SM2 Characteristics of the participants who completed the MHQ questionnaire, compared to the whole UKB cohort at baseline, and to the UK population](#SM2).
- [Table SM3**:** Prevalence of operationally defined syndromes by gender. See lettered table notes, and Appendix 2 for full case definitions](#SM3)
- [References for tables](#refs)

[Mood disorder](#Mood)

- [Figure MD1: Subclassification of mood disorder](#_Figure_MD1:_Subclassification)
- [Table MD1: Socioeconomic factors by screening status for lifetime occurrence of mood disorders](#_Table_MD1)
- [Table MD2: The occurrence of other psychiatric disorders in people positive for categories of mood disorder (comorbidities).](#_Table_MD2)
- [Table MD3: The current mental status by scores of symptoms of current disorder for categories of mood disorder](#_Table_MD3)

[Appendix 1: Members of the UK Biobank Mental Health Consortium (January 2015)](#Appendix1)

[Appendix 2: Case-control Definitions](#Appendix2)

[Appendix 3: STROBE Checklist](#Appendix5)

**Extra tables and figure**

| Table SM1: The structure of the UK Biobank mental health questionnaire “thoughts and feelings” | | | |
| --- | --- | --- | --- |
| Domain/question topic | Purpose | Source/tool | Notes about source/tool |
| A. Screening questions | To screen for presence and absence of any mental health condition. | Devised by the study team |  |
| B. Current Depression | Indicates likely presence / absence and severity of current depression. | Patient Health Questionnaire 9-question version (PHQ-9)^134^ | Maps on to criteria for DSM-IV major depressive disorder. This includes repeating the four PHQ questions asked at the baseline assessment. |
| B. Lifetime Depression | Assess lifetime history of symptoms of depression to stratify into cases and controls for genomic and other studies. | CIDI-SF (Composite International Diagnostic Interview – Short Form)^2^, depression module, lifetime version | Maps on to DSM-IV major depressive disorder. Lifetime version by Doug Levinson. Allows comparison with other cohorts in the international Psychiatric Genetics Consortium (PGC). |
| B. Lifetime manic symptoms | Identify symptoms that may indicate a bipolar affective disorder, in particular to distinguish from unipolar depression. | Devised by the study team based on CIDI questions | These questions were also included in the baseline assessment for the last one-third of UK Biobank participants^3^ |
| C. Current anxiety disorder | Indicates likely presence / absence and severity of anxiety disorder. | Generalised Anxiety Disorder Questionnaire– 7 questions (GAD-7)^1^ | A tool commonly used in research and clinical practice with PHQ-9. Maps on to DSM-IV generalised anxiety disorder, but is also raised in other anxiety disorders^1^. |
| C. Lifetime anxiety disorder | Assess lifetime occurrence of anxiety disorder. | CIDI-SF^2^, anxiety module, lifetime version | Maps on to DSM generalised anxiety disorder. Lifetime version by Doug Levinson. Allows comparison with other cohorts in the PGC. |
| D. Addictions | Assess of a variety of addictions, past and current, through prompted self-report. | Devised by the study team | Common addictions were identified from the literature: alcohol, sedatives and painkillers, illicit drugs, and behaviours such as gambling |
| E. Alcohol Use | Comprehensive assessment of patterns in alcohol use, with a view to defining misuse. | Alcohol Use Disorders Identification Test (AUDIT)^4^ | Developed by the WHO and extensively used and studied for alcohol use disorders, including hazardous and harmful drinking. |
| E. Cannabis Use | Identify cannabis use and pattern of use. | Devised by the study team | Two questions: times used and frequency used when using |
| F. Unusual experiences | Assess experience of phenomena that may be markers of psychosis. | CIDI, psychosis module, lifetime version, abridged^5^ | The CIDI lifetime version is a World Health Organisation (WHO) instrument for mental health surveys. The CIDI questions were adapted for self-report and reduced in number to as few questions as possible to tap into this theme, while making it possible to compare with the World Mental Health Surveys. |
| G. Adverse events in childhood | Identify abuse and other adverse events in childhood. | Childhood Trauma Screener – 5 item (CTS-5)^6^ | This is the short version of the Childhood Trauma Questionnaire, designed for adults to rate adverse events that may have happened in childhood^7^. |
| G. Adverse events in adult life | Identify domestic abuse and other adverse events in adult life, and lifetime trauma | Devised by the study team, based on existing questions | Using the same structure as the CTS, the questions were adapted from the national crime survey questions to identify victims of crime and adult domestic violence^8^. A short checklist of possible catastrophic trauma was also included^9^ |
| G. Post-traumatic stress disorder | Assess the occurrence of post-traumatic stress disorder. | Post-traumatic stress disorder Check List – civilian Short version (PCL-6)^10^ | Maps onto the DSM-IV criteria and is well-validated. Does not require endorsement of specific items on the trauma checklist |
| H. Self-harm and suicidal thoughts | Assess self-harm and suicidal thoughts and associated outcomes. | Devised by the study team | There were no instruments that were considered adequate, especially in terms of distinguishing between self-harm without suicidal intent and suicide attempts. The working group devised a set of questions, working with service users group on acceptability. |
| J. Subjective wellbeing | Included in response to feedback for the service user group | Devised by the study team, based on existing questions | Measures of wellbeing, two euthymic (‘positive emotion’) questions UKB baseline and one eudemonic (‘meaning’) question from the WHO-Quality Of Life (WHOQOL) ^11^. |
| K. Free-text box | To enable participants to add any further information about their mental health status. Included in response to piloting |  | In response to piloting feedback, we included a text box for the participant to elaborate on their answers and to increase the participants’ confidence in the questionnaire. |

**Table SM2** Characteristics of the participants who completed the MHQ questionnaire, compared to the whole UKB cohort at baseline, and to the UK population. Characteristics at baseline unless stated

NA = missing, not stated or not available.

See lettered table notes, and Appendix 2 for full case definitions. Numbers in superscript are references.

|  |  | UKB baseline^a^  N=502,618 | UKB MHQ^b^  N=157,366 | Illustrative population data^h^ | Population data details^c^ |
| --- | --- | --- | --- | --- | --- |
| Personal Characteristics |  |  |  |  |  |
| Age^d^ | 45-54 | 15% | 15% | 36% | Census UK 2011^12^, as proportion of people aged 45-82 |
|  | 55-64 | 30% | 33% | 30% |  |
|  | 65-74 | 44% | 45% | 22% |  |
|  | 75+ (oldest is 82^d^) | 11% | 8% | 12% |  |
| Gender | male | 46% | 43% | 48% | Census EWS 2001^13^ age 40-69 |
|  | female | 54% | 57% | 52% |  |
| Ethnicity | white | 94% | 97% | 95% | Census EWS 2001^13^ age 40-69 |
|  | black | 2% | 1% | 2% |  |
|  | asian (indian sub-continent) | 2% | 1% | 3% |  |
|  | mixed | 1% | 1% | <0.5% |  |
|  | chinese | <0.5% | <0.5% | <0.5% |  |
|  | other | 1% | 1% | 1% |  |
|  | NA | 1% | <0.5% | NA |  |
| Migrant to UK^e^ | | 9% | 7% | NA |  |
| Townsend Deprivation Score (TDS)^f^ | most deprived (TDS ≥ +2) | 16% | 12% | 30% | TDS of people invited to take part in UK Biobank, as reported in Fry et al^14^ |
|  | middle (TDS -2 to +1.99) | 32% | 31% | 34% |  |
|  | least deprived (TDS < -2) | 52% | 56% | 36% |  |
| Highest qualification | none | 17% | 7% | 38% | Census EWS 2011^12^ age 50+ |
|  | other (including vocational) | 5% | 5% | 12% |  |
|  | Secondary school equivalent | 33% | 29% | 21% |  |
|  | A-level or equivalent | 11% | 13% | 7% |  |
|  | degree | 32% | 45% | 23% |  |
|  | NA | 2% | 1% | NA |  |
| SOC job code^g^ | Higher managerial, administrative and professional | 47% | 60% | 38% | Census EW 2001^13^ age 45-64 (combined current occupation and former occupation for not working) |
|  | Intermediate occupations and small employers | 11% | 8% | 23% |  |
|  | Routine and manual occupations | 6% | 4% | 21% |  |
|  | NA | 35% | 29% | 18% |  |
| Household |  |  |  |  |  |
| Own or rent | own outright | 52% | 55% | 41% | Census EW 2001^13^ age 50-64 |
|  | own with mortgage | 36% | 38% | 40% |  |
|  | rent – social | 6% | 3% | 13% |  |
|  | rent – private | 3% | 2% | 5% |  |
|  | other | 3% | 2% | 2% |  |
| Average household income | less than £18,000 | 19% | 12% | NA |  |
|  | 18-30,000 | 22% | 21% | NA |  |
|  | 31-52,000 | 22% | 26% | NA |  |
|  | 53-100,000 | 17% | 23% | NA |  |
|  | >£100,000 | 5% | 7% | NA |  |
|  | Not stated | 14% | 10% | NA |  |
| Live with husband, wife or partner | | 72% | 75% | 77% | Census EW 2001^13^ age 50-64 |
| Health and health-behaviours |  |  |  |  |  |
| Smoking status | current | 11% | 7% | 19% | HSE 2008 age 45-64, as reported in Fry et al^14^  Number out of 4647 |
|  | former | 34% | 35% | 31% |  |
|  | never | 54% | 57% | 49% |  |
| Felt depressed in last two weeks (baseline) | “Not at all” | 73% | 77% | NA |  |
|  | “Several days” or more | 23% | 20% | NA |  |
|  | NA | 5% | 3% | NA |  |
| Report longstanding illness, disability or infirmity | Yes | 32% | 28% | 37% | Census EW 2001^13^ age 50-64 |
|  | No | 66% | 70% | 63% |  |
|  | NA | 3% | 2% | 0% |  |
| Self-report physician diagnosis | Diabetes | 5% | 3% | 5% | HSE 2009 age 45-64^15^  Number out of 1395 |
|  | Cancer | 8% | 7% | NA |  |

a) The whole UK Biobank cohort for whom information was available

b) The UK Biobank participants who completed the questionnaire in time for the data release on 8 August 2017

c) Census data from 2001 or 2011 as available for UK, EWS = England, Wales and Scotland, Eng. = England, EW = England and Wales. Where census data unavailable Health Survey for England (HSE) is used, which is a survey of around 10,000 people that through sampling and weighting of data, attempts to derive data that is representative of the population in England.

d) Age when mental health questionnaire completed, derived from date of birth. This shows evidence that UKB recruited people older than 69 years.

e) Not born in UK

f) Townsend material Deprivation Score is based on postcode areas, derived from the census data for those areas in the domains of access to a car, overcrowding of housing, lack of owner-occupation and unemployment. The tertiles are based on those used in the Fry et al. paper on socioeconomic features of UK Biobank

g) Cascot job codes, reported in nine classes in UK Biobank, arranged into three categories. UK Biobank has both directly coded and derived job codes, and we used both, but there remained significant proportion of participants with no job code. NA also includes 'never worked'.

h) Due to different patterns of missingness, percentages in this column may not add up to 100%, with shortfall being NA.

.

Table SM3**:** Prevalence of operationally defined syndromes by gender. See lettered table notes, and Appendix 2 for full case definitions.

|  |  | **Total (n=157,366)** | **Female (n=89,101)** | **Male (n=68,265)** |
| --- | --- | --- | --- | --- |
| **Lifetime history** | **Depression^a^** | 37434  (24%) | 25815  (29%) | 11619  (17%) |
|  | **Hypomania/mania^b^** | 2396  (2%) | 1288  (1%) | 1108  (2%) |
|  | **Lifetime anxiety disorder (GAD)^c^** | 11111  (7%) | 7404  (8%) | 3707  (5%) |
|  | **Unusual experience^d^** | 7803  (5%) | 4718  (5%) | 3085  (5%) |
|  | **Self-harm^e^** | 6872  (4%) | 4770  (5%) | 2102  (3%) |
| **Recent or current** | **Hazardous / harmful alcohol use^f^** | 32602 (21%) | 12175 (14%) | 20427 (30%) |
|  | **PTSD^g^** | 10064  (6%) | 6709  (8%) | 3355  (5%) |
| **Overall** | **None of above** | 86474 (55%) | 49482 (56%) | 36992 (54%) |
|  | **1+ of above** | 70892 (45%) | 39619 (44%) | 31273 (46%) |

a) Criteria met for major depressive disorder on CIDI-SF lifetime

b) Criteria met for hypomania / mania lasting for at least one week in their life

c) Criteria met for generalised anxiety disorder on CIDI-SF lifetime

d) Report potential hallucincation or delusion at any point in their life

e) Report deliberate self-harm at some point in their life, whether or not they meant to end their life

f) Criteria met for alcohol use disorder on AUDIT during the last year

g) Criteria met for post-traumatic stress disorder on PCL-6 in the last month

**References for extra tables**

1. Kroenke K, Spitzer RL, Williams JB, Löwe B. The patient health questionnaire somatic, anxiety, and depressive symptom scales: a systematic review. *General Hospital Psychiatry*. 2010; **32**(4): 345-59.

2. Kessler RC, Andrews G, Mroczek D, Ustun B, Wittchen HU. The World Health Organization composite international diagnostic interview short‐form (CIDI‐SF). *Int J Methods Psychiatr Res*. 1998; **7**(4): 171-85.

3. Smith DJ, Nicholl BI, Cullen B, Martin D, Ul-Haq Z, Evans J, et al. Prevalence and characteristics of probable major depression and bipolar disorder within UK biobank: cross-sectional study of 172,751 participants. *PLoS One*. 2013; **8**(11): e75362.

4. Reinert DF, Allen JP. The alcohol use disorders identification test: an update of research findings. *Alcoholism: Clinical and Experimental Research*. 2007; **31**(2): 185-99.

5. McGrath JJ, Saha S, Al-Hamzawi A, et al. Psychotic experiences in the general population: a cross-national analysis based on 31 261 respondents from 18 countries. JAMA psychiatry 2015; 72(7): 697-705.

6. Glaesmer H, Schulz A, Häuser W, Freyberger HJ, Brähler E, Grabe H-J. [The childhood trauma screener (CTS)-development and validation of cut-off-scores for classificatory diagnostics]. *Psychiatrische Praxis*. 2013; **40**(4): 220-6.

7. Walker EA, Gelfand A, Katon WJ, Koss MP, Von Korff M, Bernstein D, et al. Adult health status of women with histories of childhood abuse and neglect. *Am J Med*. 1999; **107**(4): 332-9.

8. Khalifeh H, Oram S, Trevillion K, Johnson S, Howard LM. Recent intimate partner violence among people with chronic mental illness: findings from a national cross-sectional survey. *The British Journal of Psychiatry*. 2015; **207**(3): 207-12.

9. Frissa S, Hatch SL, Fear NT, Dorrington S, Goodwin L, Hotopf M. Challenges in the retrospective assessment of trauma: comparing a checklist approach to a single item trauma experience screening question. *BMC psychiatry*. 2016; **16**(1): 20.

10. Wilkins KC, Lang AJ, Norman SB. Synthesis of the psychometric properties of the PTSD checklist (PCL) military, civilian, and specific versions. *Depression and anxiety*. 2011; **28**(7): 596-606.

11. Forgeard MJ, Jayawickreme E, Kern ML, Seligman ME. Doing the right thing: Measuring wellbeing for public policy. *International Journal of Wellbeing*. 2011; **1**(1).

12. Office for National Statistics, National Records of Scotland, Northern Ireland Statistics and Research Agency. 2011 Census aggregate data. UK Data Service. This information is licensed under the terms of the Open Government Licence [http://www.nationalarchives.gov.uk/doc/open-government-licence/version/2]. 2017.

13. Office for National Statistics. 2001 Census aggregate data. UK Data Service.: 2011

14. Fry A, Littlejohns TJ, Sudlow C, Doherty N, Adamska L, Sprosen T, et al. Comparison of sociodemographic and health-related characteristics of UK Biobank participants with the general population. *Am J Epidemiol*. 2017: 10.1093/aje/kwx246.

15. National Centre for Social Research, University College London. Health Survey for England 2009. UK Data Archive, 2015.

**Supplementary material: Mood disorder**

Using data on participants’ history of depression and hypomania/mania, we divided the cohort according to five likely mood disorder categories as shown in Figure MD1. To be classified as having met DSM criteria for depression, participant needed to have five symptoms including depressed mood and/or anhedonia, for most of the time for two weeks. To be classified as having hypomanic / manic symptoms, participants needed to have four symptoms including high elevated mood or five symptoms with irritable mood. Subthreshold depression was used when participants had depressed mood and/or anhedonia most of the time for two weeks, but didn’t have five symptoms in total; also, if they had a diagnosis of depression but had not met diagnostic criteria or if they appeared to have current depression on the PHQ despite not meeting diagnostic criteria.

While we would expect most participants with DSM IV bipolar affective disorder type I to be categorised as such, bipolar disorder without depression (i.e. recurrent mania) will be included in the “no depression” category. Participants who have experienced less disruptive symptoms of hypomania (including many with DSM IV bipolar affective disorder type II) will be included in the single depression or recurrent depression categories.

Table MD1 shows the socio-demographic and risk factors by likely mood disorder categories. Respondents with unipolar depression appear to be more likely to be female, an association not shared with those with bipolar disorder. Participants with likely bipolar disorder were younger, and resided in more deprived areas on average. Respondents with subthreshold depressive symptoms or any likely mood disorder group reported more lifetime adverse events, but these were highest for those respondents with bipolar disorder. There are also elevated rates of neuroticism, loneliness and longstanding illness or disability among subthreshold, unipolar depression and bipolar disorder.

Table MD2 shows that the other operationally defined syndromes are more common amongst respondents with a likely mood disorder. Table MD3 reports current mental state of the participants from the average score on the scales included in the MHQ, again split by likely mood disorder, which shows some variation between the groups. Notably, wellbeing appears to be affected by depressive symptoms, whether or not reaching diagnostic criteria.

# Figure MD1: Subclassification of mood disorder


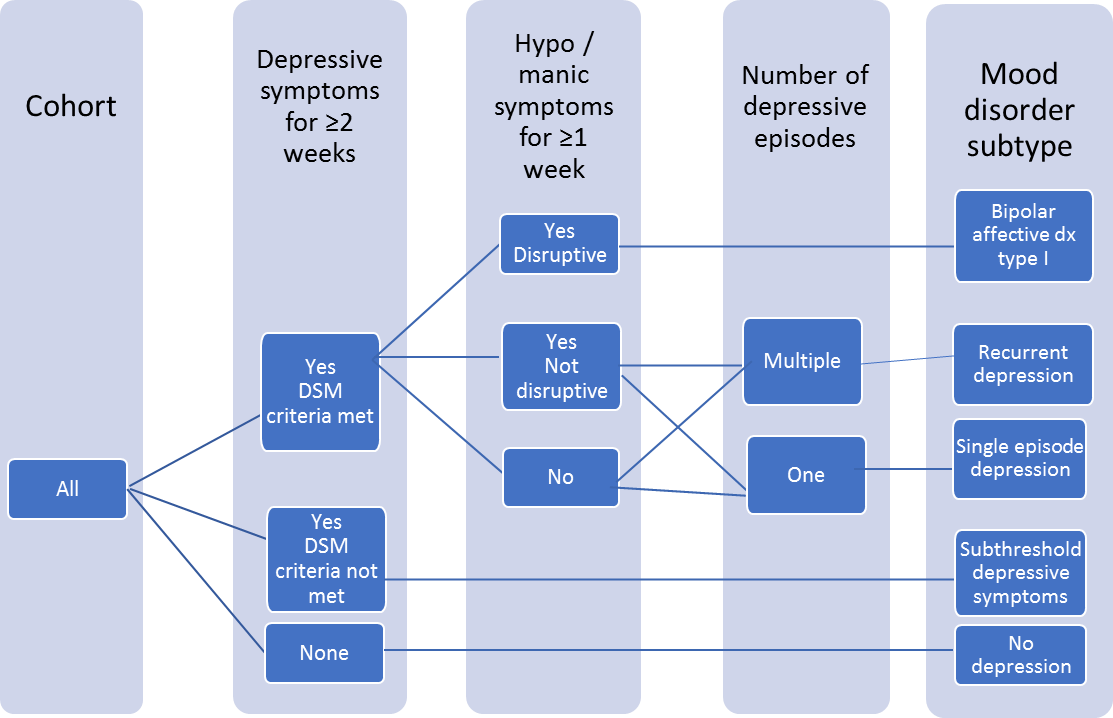


# Table MD1

Socioeconomic factors by screening status for lifetime occurrence of mood disorders broken down into exclusive categories. See figure MD1 for categorisation details.

|  |  | **No depression^a^**  **(n=88,650)** | **Subthreshold depressive symptoms^b^ (n=27,207)** | **Lifetime depression^c^, single episode (n=14,683)** | **Lifetime depression^c^, recurrent^d^ (n=21,187)** | **Bipolar affective disorder type 1^e^ (n=931)** |
| --- | --- | --- | --- | --- | --- | --- |
| **Personal Characteristics** |  |  |  |  |  |  |
| **Age^f^** | 45-54 | 11332 (13%) | 4298 (16%) | 2454 (17%) | 4334 (20%) | 228 (24%) |
|  | 55-64 | 26755 (30%) | 8894 (33%) | 5450 (37%) | 8709 (41%) | 417 (45%) |
|  | 65-74 | 42800 (48%) | 11972 (44%) | 5939 (40%) | 7297 (34%) | 261 (28%) |
|  | 75+ (oldest is 82) | 7763 (9%) | 2043 (8%) | 840 (6%) | 847 (4%) | 25 (3%) |
| **Gender** | female | 44831 (51%) | 16105 (59%) | 9902 (67%) | 15000 (71%) | 532 (57%) |
| **Ethnicity** | white | 85731 (97%) | 26185 (96%) | 14299 (97%) | 20509 (97%) | 892 (96%) |
| **Townsend Deprivation Score^g^** | most deprived (TDS ≥ +2) | 9204 (10%) | 3927 (14%) | 1800 (12%) | 3518 (17%) | 201 (22%) |
| **Highest qualification** | degree | 40597 (46%) | 11648 (43%) | 6582 (45%) | 9665 (46%) | 425 (46%) |
| **Housing tenure** | rent^h^ | 3024 (3%) | 1825 (7%) | 796 (5%) | 1869 (9%) | 155 (17%) |
| **Risk factors** |  |  |  |  |  |  |
| **Neuroticism score^i^** | mean (SD) | 2.8 (2.6) | 5.0 (3.2) | 4.5 (3.1) | 6.2 (3.3) | 3.8 (3.1) |
| **Adverse life experiences** | childhood screen^j^ | 34223 (39%) | 13907 (51%) | 7283 (50%) | 12823 (61%) | 638 (69%) |
|  | adult screen^k^ | 38802 (44%) | 15962 (59%) | 8406 (57%) | 14361 (68%) | 685 (74%) |
|  | trauma exposure^l^ | 40761 (46%) | 14675 (54%) | 8052 (55%) | 13049 (62%) | 665 (71%) |
| **Social connection^,m^** | loneliness | 1704 (2%) | 1715 (6%) | 621 (4%) | 1602 (8%) | 94 (10%) |
|  | social isolation | 5927 (7%) | 2651 (10%) | 1287 (9%) | 2336 (11%) | 126 (14%) |
| **Illness^i^** | longstanding illness, disability or infirmity | 19846 (22%) | 9046 (33%) | 4443 (30%) | 8072 (38%) | 503 (54%) |
| **Health-behaviours** |  |  |  |  |  |  |
| **Smoking status^i^** | current | 5077 (6%) | 2334 (9%) | 1223 (8%) | 2171 (10%) | 158 (17%) |
| **Cannabis use** | ever daily | 838 (1%) | 500 (2%) | 246 (2%) | 584 (3%) | 63 (7%) |
| **Physical activity^i^** | moderate activity ≥3 times a week | 31899 (36%) | 10392 (38%) | 5374 (37%) | 8024 (38%) | 345 (37%) |

1. Does not meet criteria for major depressive disorder or subthreshold depressive symptoms
2. Does not meet criteria for major depressive disorder, but features suggestive of increased symptoms compared to general population
3. Criteria met for major depressive disorder on CIDI-SF lifetime
4. Reports more than one episode or “too many to recall”
5. Criteria met for major depressive disorder and mania
6. Age when mental health questionnaire released, derived from date of birth
7. Townsend material Deprivation Score is based on postcode areas^14^
8. Collapsed from categories in SM1
9. From baseline assessment 2006-10
10. Criteria met for possible abuse or neglect on Childhood Trauma Screener
11. Criteria met for stressful situations, including abusive relationships and money problems, as an adult
12. Reports one or more of six situations known to be triggers for trauma-related mental disorders
13. There is some overlap between the adult adverse life experiences screen and loneliness screen, which both ask about confiding relationships: adult screen includes lack of confiding relationship over the adult lifetime; loneliness includes lack of confiding relationship at the time of baseline assessment

# Table MD2

The occurrence of other psychiatric disorders in people positive for categories of mood disorder (comorbidities). See lettered table notes, and Appendix 2 for full case definitions

|  | No depression^a^  (n=88,650) | Subthreshold depressive symptoms^b^ (n=27,207) | Lifetime depression^c^, single episode (n=14,683) | Lifetime depression^c^, recurrent^d^ (n=21,187) | Bipolar affective disorder type 1^e^ (n=931) |
| --- | --- | --- | --- | --- | --- |
| Lifetime anxiety disorder (GAD)^f^ (n=11,111) | 746 (1%) | 1806 (7%) | 1770 (12%) | 6035 (28%) | 449 (48%) |
| Current hazardous/harmful alcohol^g^ (n=32,602) | 17495 (20%) | 5991 (22%) | 2874 (20%) | 4875 (23%) | 266 (29%) |
| Current PTSD^h^ (n=10,064) | 705 (1%) | 2776 (10%) | 1122 (8%) | 4684 (22%) | 366 (39%) |
| Lifetime unusual experiences^i^ (n=7,803) | 2314 (3%) | 1642 (6%) | 904 (6%) | 2263 (11%) | 329 (35%) |
| Lifetime hypomania/mania^j^ (n=2,396) | 351 (<0.5%) | 454 (2%) | 123 (1%) | 496 (2%) | 931 (100%) |
| Overall |  |  |  |  |  |
| None (n=107,347) | 68550 (77%) | 18535 (68%) | 9745 (66%) | 10517 (50%) | NA |
| At least one (n=45,310) | 20099 (23%) | 8672 (32%) | 4938 (34%) | 10670 (50%) | 931 (100%) |
| Multiple (n=5,910) | 775 (1%) | 1102 (4%) | 676 (5%) | 2658 (13%) | 699 (75%) |

1. Does not meet criteria for major depressive disorder or subthreshold depressive symptoms
2. Does not meet criteria for major depressive disorder, but features suggestive of increased symptoms compared to general population
3. Criteria met for major depressive disorder on CIDI-SF lifetime
4. Reports more than one episode or “too many to recall”
5. Criteria met for major depressive disorder and mania
6. Criteria met for generalised anxiety disorder on CIDI-SF lifetime
7. Criteria met for alcohol use disorder on AUDIT during the last year
8. Criteria met for post-traumatic stress disorder on PCL-6 in the last month
9. Reported hallucincation and/or delusion at any point in their life
10. Criteria met for hypomania / mania lasting for at least one week

# Table MD3

The current mental status by scores of symptoms of current disorder for categories of mood disorder. See lettered table notes, and Appendix 2 for full case definitions.

| **Mean (SD) Median** | **No depression^a^** | **Subthreshold depressive symptoms^b^ (n=27,207)** | **Lifetime depression^c^, single episode (n=14,683)** | **Lifetime depression^c^, recurrent^d^ (n=21,187)** | **Bipolar affective disorder type 1^e^ (n=931)** |
| --- | --- | --- | --- | --- | --- |
|  | **(n=88,650)** |  |  |  |  |
| **Depression score PHQ-9** | 1.1 (1.2) 1^f^ | 5.1 (4.3) 5 | 3.1 (3.6) 2 | 5.9 (5.5) 4 | 7.9 (7.0) 6 |
| scale 0-36, high = more depressed |  |  |  |  |  |
| **Anxiety score GAD-7** | 1.0 (1.8) 0 | 3.5 (4.0) 2 | 2.5 (3.5) 1 | 4.7 (4.9) 4 | 6.2 (6.1) 5 |
| scale 0-28, high = more anxious |  |  |  |  |  |
| **Alcohol use disorder score AUDIT** | 4.8 (3.8) 4 | 5.1 (4.6) 4 | 4.8 (4.2) 4 | 5.2 (5.1) 4 | 6.1 (6.3) 4 |
| scale 0-42, high = more hazardous |  |  |  |  |  |
| **Wellbeing score** | 13.3 (1.6) 13 | 11.9 (2.1) 12 | 12.5 (2.0) 13 | 11.4 (2.3) 12 | 11.0 (2.7) 11 |
| scale 3-22, high = better wellbeing |  |  |  |  |  |

1. Does not meet criteria for major depressive disorder or subthreshold depressive symptoms
2. Does not meet criteria for major depressive disorder, but features suggestive of increased symptoms compared to general population
3. Criteria met for major depressive disorder on CIDI-SF lifetime
4. Reports more than one episode or “too many to recall”
5. Criteria met for major depressive disorder and mania
6. A PHQ-9 score above 5 would exclude the participant from the “No depression” category

# Appendix 1:

**Members of the UK Biobank Mental Health Consortium, January 2015**

**(institutions as per 2015).**

**Chair:** Matthew Hotopf, Psychological Medicine and SLaM/Institute of Psychiatry, Psychology and Neuroscience Biomedical Research Centre, Kings College London

**Steering Group Members**

· Gerome Breen, MRC Social, Genetic & Developmental Psychiatry Centre, Kings College London

· Katrina Davis, Research Assistant to Matthew Hotopf

· Elaine Fox, Oxford Centre for Emotions and Affective Neuroscience, Department of Experimental Psychology, University of Oxford

· Louise Howard, Section of Women’s Mental Health, Health Service and Population Research, Kings College London

· Ann John, College of Medicine, Swansea University Medical School and National Centre for Mental Health

· Rose McCabe, Clinical Communication and Mental Health, University of Exeter

· Andrew McIntosh, Division of Psychiatry, University of Edinburgh and Scottish Mental Health Research Network

· Daniel Smith, Institute of Health and Wellbeing, University of Glasgow

· Stan Zammit, MRC Centre for Neuropsychiatric Genetics and Genomics, Cardiff University

**Members**

· Naomi Allen, University of Oxford and UK Biobank

· David Batty, Centre for Cognitive Ageing and Cognitive Epidemiology, University of Edinburgh and University College London

· Cyrus Cooper, MRC Lifecourse Epidemiology Unit, University of Southampton

· Tim Croudace, Centre for Health and Population Sciences, University of York

· Ian Deary, Centre for Cognitive Ageing and Cognitive Epidemiology, University of Edinburgh

· Christopher Dickens, Institute of Health Service Research, University of Exeter

· Klaus Ebmeier, Division of Psychiatry, University of Oxford

· Seena Fazel, Division of Psychiatry, University of Oxford

· Robin Flaig, Assistant to Cathie Sudlow for UK Biobank

· Jonathon Flint, Wellcome Trust Centre for Human Genetics, University of Oxford

· John Gallacher, University of Oxford and MRC Dementias Platform UK

· Simon Gilbody, Centre for Health and Population Sciences, University of York

· Hazel Inskip, MRC Lifecourse Epidemiology Unit, University of Southampton

· Tony Kendrick, Department of Primary Care, University of Southampton

· David Kingdon, University of Southampton and Mental Health Foundation

· William Lee, Plymouth University Peninsula Schools of Medicine and Dentistry & Devon Partnership NHST.

· Glyn Lewis, Clinical Trials and Applied Epidemiology in Psychiatry, University College London

· Donald Lyall, Institute of Health and Wellbeing, University of Glasgow

· Donald MacIntyre, Division of Psychiatry, University of Edinburgh

· Susan McAndrew, Mental Health and Wellbeing Unit, University of Salford

· Peter McGuffin, MRC Social, Genetic & Developmental Psychiatry Centre, Kings College London

· Irwin Nazareth, Primary Care and Public Health, University College London

· Barbara Nicholl, Institute of Health and Wellbeing, University of Glasgow

· Michael O’Donovan, Institute of Psychological Medicine and Clinical Neuroscience, Cardiff University

· Jolanta Opacka-Juffry, Health Sciences Research Unit, Rohampton University

· David Osborn, Division of Psychiatry, University College London

· Michael Owen, Institute of Psychological Medicine and Clinical Neuroscience, Cardiff University

· Carmine Pariante, Department of Psychological Medicine, Kings College London

· Marcus Richards, Population Health Sciences, University College London

· Theresa Rushe, Department of Psychology, Queens University Belfast

· Valerie Shanks-Pepper, Medical Directorate, NHS England

· Stephen Stansfeld, Centre for Psychiatry, Queen Mary University of London

· Geraldine Strathdee, University College London and NHS England

· Cathie Sudlow, University of Edinburgh and UK Biobank

· Elizabeth Tunbridge, Department of Psychiatry, University of Oxford

· Scott Weich, Mental Health and Wellbeing, University of Warwick

· Peter Woodruff, Academic Clinical Psychiatry, University of Sheffield

· Allan Young, Department of Psychological Medicine, Kings College London

**International Contributors**

· Brenda Penninx, Professor of Psychiatric Epidemiology, VU University Amsterdam, NL

· Douglas Levinson, Professor of Psychiatry, Stanford University, USA

· Kenneth Kendler, Professor and Eminent Scholar Psychiatry, Virginia Commonwealth University, USA

· Hans Grabe, Professor in the Department of Psychiatry and Psychotherapy, Greifswald University, GL

John McGrath, Niels Bohr Professor, National Centre for Register-based Research, Aarhus University, Denmark & University of Queensland, AUS

# Appendix 2: Case Criteria Derived from the UK Biobank Mental Health Questionnaire

Tables refer to the tables in results section and supplementary material of Davis et al. *Mental Health in UK Biobank – development, implementation and results from an online questionnaire completed by 157,366 participants.*

Control definitions are fairly stringent, for applications where higher certainty that control groups do not contain cases is requred. For the above paper, non-caseness was used rather than the control definitions below, and this may be preferred for many purposes.

| Tables | **Disorder / Exposure** | **Rule in English** | **Fields and codes** | **Notes and references** |
| --- | --- | --- | --- | --- |
| Mood disorder | | | | |
| All | Depression | **Case: Depression ever.**  At least one core symptom of depression, most or all of the day on most or all days for a two week period, with at least five depressive symptoms that represent a change from usual occurring over the same time-scale, with some or a lot of impairment. | Persistent sadness (20446) = Yes OR Loss of interest (20441) = Yes  AND  How much of day (20436) = Most of day or All day long  AND  Did you feel this way (20439) = Almost every day or Every day  AND  Impairment (20440) = Somewhat or A lot  AND  Total number of symptoms endorsed (core and others) >= 5   - Persistent sadness (core) 20446; Loss of interest (core) 20441; Tired or low energy 20449; Gain or loss of weight 20536 = Gain, Loss or Gain and loss; Sleep change 20532; Trouble concentrating 20435; Feeling worthless 20450; Thinking about death 20437 | CIDI-SF (Composite International Diagnostic Interview – Short Form), depression module, lifetime version. Scored based on DSM definition of major depressive disorder  *Kessler RC, Andrews G, Mroczek D, Ustun B, Wittchen HU. The World Health Organization composite international diagnostic interview short‐form (CIDI‐SF). Int J Methods Psychiatr Res. 1998;7(4):171-85.* |
| MD1, MD2, MD3 | Depression | **Case: Subthreshold depressive symptoms ever.**  Does not meet diagnostic criteria for depression on the CIDI, but has at least one of: (i) endorses persistent depression or anhedonia on CIDI; (ii) PHQ9 (current depressive symptoms) is over threshold for mild depression; (iii) reports clinician diagnosis of depression | NOT Case {depression ever}  AND  ((reported diagnosis of depression 20544 or 20002)  OR  Core symptoms from above  OR  PHQ score >5) | Case plus control plus subthreshold should include all participants with valid responses  Subthreshold symptoms may have clinical significance  *National Institute for Health and Clinical Excellence. Depression in adults: recognition and management. NICE Clinical Guideline CG90 (available at https://www.nice.org.uk/guidance/cg90) 2009 (updated 2016).* |
| nil | Depression | **Control: Depression ever.**  Not endorsing depression or screening positive on PHQ or CIDI | NOT (reported diagnosis of depression 20544 or 20002)  AND  NOT Core symptoms from above  AND  PHQ score ≤5 | Case plus control plus subthreshold should include all participants with valid responses. By excluding subthreshold symptoms, we can be confident that this group has not experienced a classical depressive episode |
| MD1, MD2, MD3 | Depression | **Case: Depression single episode.** | Case {depression ever}  AND  Number of episodes (20442)=1  AND  NOT case {bipolar type I}  Excluded if number of episodes missing or bipolar state missing | Single episode, recurrent depression and bipolar type I should include all depression cases with valid responses |
| MD1, MD2, MD3 | Depression | **Case: Recurrent depression.** | {depression ever}  AND  Number of episodes (20442) >1 or -999 (too many to count)  AND  NOT case {bipolar type I}  Excluded if number of episodes missing or bipolar state missing | Single episode, recurrent depression and bipolar type I should include all depression cases with valid responses |
| nil | Depression | ***Variant: Depression single episode triggered by loss*** | {depression single episode}  AND  worst depression start within two months of traumatic event (20447) = yes | Cases of single episode triggered by loss could be selectively excluded for some analyses, although likely to exclude some true cases of major depressive episode |
| MD3 | Depression | **Score: PHQ-9.**  score items 0-4 and sum  (Little interest or pleasure in doing things 20514, Feeling down, depressed, or hopeless 20510, Trouble sleeping 20517, Feeling tired 20519, Poor appetite or overeating 20511, Feeling bad about yourself 20507, Trouble concentrating 20508, Moving or speaking slowly or fidgety or restless 20518, Thoughts that you would be better off dead 20513) | (“20514, 20510, 20517, 20519, 20511, 20507, 20508, 20518, 20513”) (subtract 9 if items scored 1-5)  If value missing, count as “0” when scoring 0-4 | *Kroenke K, Spitzer RL, Williams JB, Löwe B. The patient health questionnaire somatic, anxiety, and depressive symptom scales: a systematic review. Gen Hosp Psychiatry. 2010;32(4):345-59.* |
| nil | Depression | **Case: Current depression.**  PHQ +ve and CIDI+ve  Reports symptoms in the last two weeks that have bothered them. Current depression is indicated by five or more items marked to bother at or above a certain intensity: “more than half of days” for first eight items, “some days” for last item. | {depression ever}  AND  Total symptoms endorsed as occurring more than half days (or some or more days for last item) ≥ 5   - Little interest or pleasure in doing things 20514, Feeling down, depressed, or hopeless 20510, Trouble sleeping 20517, Feeling tired 20519, Poor appetite or overeating 20511, Feeling bad about yourself 20507, Trouble concentrating 20508, Moving or speaking slowly or fidgety or restless 20518, Thoughts that you would be better off dead 20513 | For identifying likely depression, can use “diagnostic algorithm” based on DSM criteria, alternatively total score. This is using “diagnostic algorithm”  *Manea L, Gilbody S, McMillan D. Optimal cut-off score for diagnosing depression with the Patient Health Questionnaire (PHQ-9): a meta-analysis. CMAJ. 2012;184(3):E191-E6* |
| nil | Depression | **Control: Current depression.**  PHQ score ≤5 | PHQ score ≤5 | A score of above 5 on PHQ can be used as a cut-off for mild depression. Therefore this control group excludes people with possible mild depression, as well as those who meet full criteria in the diagnostic algorithm.  *Manea L, Gilbody S, McMillan D. Optimal cut-off score for diagnosing depression with the Patient Health Questionnaire (PHQ-9): a meta-analysis. CMAJ. 2012;184(3):E191-E6* |
| nil | Depression | ***Variant: Current severe depression***  *As current depression (above) with PHQ score > 15* | {depression current}  AND  PHQ score >15 | *Manea L, Gilbody S, McMillan D. Optimal cut-off score for diagnosing depression with the Patient Health Questionnaire (PHQ-9): a meta-analysis. CMAJ. 2012;184(3):E191-E6* |
| 2, MD2 | Mania | **Symptoms: Hypomania / Mania.**  Endorses features of hypomania / mania lasting for a week or more, whether or not they were disruptive, and whether or not a depression ever case. Requires “High-hyper” plus three other symptoms or “Irritable” plus four other symptoms | High/Hyper 20501 = 01 OR Irritable 20502 = 01  AND  Four features from:   - High/Hyper 20501; Active 20548(01); Talkative 20548(02); Less sleep 20548(03); Creative/ideas 20548(04); Restless 20548(5); Confident 20548(6); Thoughts racing 20548(7); Easily distracted 20548(8)   AND  Duration 20492 = A week or more | Based on DSM-IV definition of hypo/mania. This includes likely cases of bipolar affective disorder type I, possible bipolar type II (where symptoms last a week), recurrent mania without clear depression, and antidepressant-induced symptoms of hypomania / mania.  *Smith DJ, Nicholl BI, Cullen B, Martin D, Ul-Haq Z, Evans J, et al. Prevalence and characteristics of probable major depression and bipolar disorder within UK biobank: cross-sectional study of 172,751 participants. PLoS One. 2013;8(11):e75362* |
| 3,  MD1, MD2,MD3 | Mania | **Case: Bipolar affective disorder type I.**  Ever manic/hyper or irritable, plus at least three other features (four if never manic/hyper), plus duration a week or more, plus symptoms caused significant problems. Requires also to be case for depression ever. | Case {depression ever}  AND  High/Hyper 20501 = 01 OR Irritable 20502 = 01  AND  Four features from:   - High/Hyper 20501; Active 20548(01); Talkative 20548(02); Less sleep 20548(03); Creative/ideas 20548(04); Restless 20548(5); Confident 20548(6); Thoughts racing 20548(7); Easily distracted 20548(8)   AND  Duration 20492 = A week or more  AND  Symptoms caused problem 20493 = yes | Case for depression is not required in DSM-IV diagnostic criteria, but is added here to improve the positive predictive value of the test (see text and references). This definition does not exclude antidepressant-induced mania.  *Cerimele et al. The prevalence of bipolar disorder in primary care samples: a systematic review, General Hospital Psychiatry 36 (2014) 19-25*  *Carvalho, A. F., Y. Takwoingi, et al. (2015). "Screening for bipolar spectrum disorders: a comprehensive meta-analysis of accuracy studies." Journal of affective disorders* ***172****: 337-346* |
| nil | Mania | ***Variant: Case bipolar type II***  *As above, without disruption from symptoms* | *Case {depression ever}*  *AND*  *High/Hyper 20501 = 01 OR Irritable 20502 = 01*  *AND*  *Four features as above*  *AND*  *Duration 20492 = A week or more* | There is less agreement over the definition of bipolar affective disorder type II. DSM-IV criteria require symptoms for four days or more. Here is one week, so could be predicted to miss some cases. |
| nil | Mania | **Control: Hypomania / Mania**  Not included in hypomania / mania symptoms, nor categorised as bipolar on last UKB classification, nor self-reported bipolar | NOT {hypomania/mania}  AND  NOT {categorised bipolar on last UKB categorisation 20126 = 1 or 2}  AND  NOT {self-reported bipolar 20544=10} |  |
| Anxiety | | | | |
| 2,3, MD2 | GAD | **Case: GAD Ever.**  Excessive worrying about a number of issues, occurring most days for six months and difficult to control, with three or more somatic symptoms and functional impairment. | Worried tense of anxious (20421) = Yes  AND  Duration (20420) >= 6 months or All my life  AND  Most days (20538) = Yes  AND  Excessive: More than most (20425) OR Stronger than most (20542)  AND  Number of issues: More than one thing (20543) OR Different worries (20540)  AND  Difficult to control: Difficult to stop worrying (20541) OR Couldn’t put it out of mind (20539) OR Difficult to control (20537)  AND  Functional impairment: Role interference (20418) = Some or A lot  AND  3 somatic symptoms out of:  Restless. 20426; Keyed up or on edge. 20423; Easily tired. 20429; Having difficulty keeping your mind on what you were doing. 20419; More irritable than usual. 20422; Having tense, sore, or aching muscles. 20417; Often having trouble falling or staying asleep. 20427 | CIDI-SF (Composite International Diagnostic Interview – Short Form), GAD module, lifetime version. Scored based on DSM definition of GAD  *Kessler RC, Andrews G, Mroczek D, Ustun B, Wittchen HU. The World Health Organization composite international diagnostic interview short‐form (CIDI‐SF). Int J Methods Psychiatr Res. 1998;7(4):171-85.*  *National Institute for Health and Clinical Excellence. Generalised anxiety disorder and panic disorder in adults: management. NICE Clinical Guideline CG113 (available at https://www.nice.org.uk/guidance/cg113) 2011* |
| nil | GAD | **Control: GAD ever.**  Not meeting criteria for GAD ever nor scoring over low cut-off for GAD-7 | NOT case {GAD ever}  AND  GAD-7 score < 5 | Excluding those that screen positive for mild anxiety means that there is greater confidence that this group have not had anxiety disorder |
| MD3 | GAD | **Score: GAD-7**  Score 0-3 and sum  a) Feeling nervous, anxious or on edge 20506  b) Not being able to stop or control worrying 20509  c) Worrying too much about different things 20520  d) Trouble relaxing 20515  e) Being so restless that it is hard to sit still 20516  f) Becoming easily annoyed or irritable 20505  g) Feeling afraid as if something awful might happen 20512 | Sum {Feeling nervous, anxious or on edge 20506, Not being able to stop or control worrying 20509, Worrying too much about different things 20520, Trouble relaxing 20515, Being so restless that it is hard to sit still 20516, Becoming easily annoyed or irritable 20505, Feeling afraid as if something awful might happen 20512} 0,1,2,3  (nb in biobank coded 1-4, subtract 7 to adjust)  If item missing, score 0 when scoring 0-3 | *Kroenke K, Spitzer RL, Williams JB, Löwe B. The patient health questionnaire somatic, anxiety, and depressive symptom scales: a systematic review. Gen Hosp Psychiatry. 2010;32(4):345-59* |
| nil | GAD | **Case: Current anxiety.**  GAD-7 score ≥10 and case GAD ever | Case {GAD ever}  AND  GAD-7 score ≥10  Where each item scored 0-3 | Can be scored with cut-offs for mild, moderate and severe, with cut-offs at 5, 10 and 15. 10 chosen to represent moderate.  *Kroenke K, Spitzer RL, Williams JB, Löwe B. The patient health questionnaire somatic, anxiety, and depressive symptom scales: a systematic review. Gen Hosp Psychiatry. 2010;32(4):345-59* |
| nil | PTSD | **Score: PCL-6**  Sum of scores on questions representing the core symptoms of PTSD  Score 1-5 and sum  [20497Repeated disturbing thoughts of stressful experience in past month](http://biobank.ctsu.ox.ac.uk/crystal/field.cgi?id=20497)  [20498Felt very upset when reminded of stressful experience in past month](http://biobank.ctsu.ox.ac.uk/crystal/field.cgi?id=20498)  [20495Avoided activities or situations because of previous stressful experience in past month](http://biobank.ctsu.ox.ac.uk/crystal/field.cgi?id=20495)  [20496Felt distant from other people in past month](http://biobank.ctsu.ox.ac.uk/crystal/field.cgi?id=20496)  [20494Felt irritable or had angry outbursts in past month](http://biobank.ctsu.ox.ac.uk/crystal/field.cgi?id=20494)  20508 Trouble concentrating (scored 1-4) | Sum {[20497Repeated disturbing thoughts of stressful experience in past month](http://biobank.ctsu.ox.ac.uk/crystal/field.cgi?id=20497), [20498Felt very upset when reminded of stressful experience in past month](http://biobank.ctsu.ox.ac.uk/crystal/field.cgi?id=20498), [20495Avoided activities or situations because of previous stressful experience in past month](http://biobank.ctsu.ox.ac.uk/crystal/field.cgi?id=20495), [20496Felt distant from other people in past month](http://biobank.ctsu.ox.ac.uk/crystal/field.cgi?id=20496), [20494Felt irritable or had angry outbursts in past month](http://biobank.ctsu.ox.ac.uk/crystal/field.cgi?id=20494)} 1,2,3,4,5 + {20508 Trouble concentrating} 1,2,3,4  (nb biobank coded 0-4, subtract 5 to adjust) | Using PHQ item for concentration, scores out of 29 (conventionally scores out of 30), and will make it slightly harder to reach conventional threshold.  *Lang AJ, Stein MB. An abbreviated PTSD checklist for use as a screening instrument in primary care. Behaviour research and therapy. 2005;43(5):585-94* |
| 2, MD2 | PTSD | **Case: PTSD.**  PCL-6 sum of scores 14 or greater is positive screen | ([20497Repeated disturbing thoughts](http://biobank.ctsu.ox.ac.uk/crystal/field.cgi?id=20497) + [20498Felt very upset when reminded](http://biobank.ctsu.ox.ac.uk/crystal/field.cgi?id=20498) + [20495Avoided activities or situations](http://biobank.ctsu.ox.ac.uk/crystal/field.cgi?id=20495) + [20496Felt distant](http://biobank.ctsu.ox.ac.uk/crystal/field.cgi?id=20496) + [20494Felt irritable or had angry outbursts](http://biobank.ctsu.ox.ac.uk/crystal/field.cgi?id=20494) + 20508 Trouble concentrating)>13 | Does not currently require catastrophic trauma, but refers to “stressful event” in the text of the questions as this is not an exhaustive list of possible trauma. |
| nil | PTSD | **Control: PTSD.**  PCL-6 sum of scores 13 or less is positive screen. Include those who do not complete PCL-6 due to stop rule. | Answered ([20497Repeated disturbing thoughts](http://biobank.ctsu.ox.ac.uk/crystal/field.cgi?id=20497) + [20498Felt very upset when reminded](http://biobank.ctsu.ox.ac.uk/crystal/field.cgi?id=20498) + [20495Avoided activities or situations](http://biobank.ctsu.ox.ac.uk/crystal/field.cgi?id=20495))  AND  NOT {Case: PTSD} |  |
| Other symptoms | | | | |
| 2,3, MD2 | Unusual experiences | **Symptom: Unusual experience.**  Endorsed possible hallucination or delusion | Heard unreal voice 20463 = yes  OR  Saw unreal vision 20471 = yes  OR  Believed unreal conspiracy 20468 = yes  OR  Believed unreal communication or signs 20474 = yes | Adapted by group from CIDI questions  *Nuevo R, Chatterji S, Verdes E, Naidoo N, Arango C, Ayuso-Mateos JL. The Continuum of Psychotic Symptoms in the General Population: A Cross-national Study. Schizophrenia Bulletin. 2012;38(3):475-85* |
| nil | Unusual experiences | **Symptom: Recent unusual experience.**  Reports hallucination or delusion in the last year | Frequency in last year 20467>0 |  |
| nil | Unusual experiences | **Control: Unusual experience.**  Not endorsing psychotic illness or reporting symptoms | NOT Endorsed diagnosis 20544 of schizophrenia [2] or other psychotic illness [3]  AND  NOT {ever hallucination} OR {ever delusion} |  |
| nil | Self-harm | **Case: Life not worth living.**  Ever felt life not worth living | 20479 life NWL = yes (1 or 2) |  |
| 2, MD2 | Self-harm | **Case: Self harm.**  Ever harmed self, whether or not meant to die | 20480 Self harmed = Yes | Self harm is further divided by whether have ever self-harmed with intent to die (question 20483) |
| nil | Self-harm | **Case: Non-suicidal self-harm**  Self-harm without intention to end life | 20480 Self harmed = Yes  20483 Attempted suicide = No |  |
| nil | Self-harm | **Case: Suicide attempt**  Ever harmed self with intent to end life | 20483 Attempted suicide = Yes | Does not rule out that on different occasion engaged in NSSI behaviour. |
| Alcohol and addiction | | | | |
| MD3 | Alcohol | **Score: AUDIT**  Asks about “in the last year” apart from last two questions.  (Note coding on UKB is from 1-5, so requires adjustment)  Sum individual scores | PART 1 Hazard: Frequency (scored 0-4) 20414, typical drinks (score 0-4) 20403, six or more drinks (scored 0-4) 20416  PART 2 Dependence: Unable to stop (scored 0-4) 20413, failed to do what expected due to drinking (scored 0-4) 20407, needed to drink first thing (scored 0-4) 20412  PART 3 Harm: Guilt due to drinking (scored 0-4) 20409, unable to remember due to drink (scored 0-4) 20408, injury due to drinking ever (scored 0,2,4) 20411, advice to cut down ever (scored 0,2,4) 20405 | Can be scored using algorithm or cut-offs, with more literature on the latter approach. Using cut-off of 8 is to indicate likelihood of moderate severity, 16 indicates severe, and lower cut-offs have been used to identify hazardous drinking (as opposed to drinking already causing harm).  *Reinert, D. F. and J. P. Allen (2007). "The alcohol use disorders identification test: an update of research findings." Alcoholism: Clinical and Experimental Research 31(2): 185-199* |
| 2, MD2 | Alcohol | **Case: Hazardous / Harmful Alcohol Use.**  Alcohol use disorder of moderate severity (also called hazardous/harmful drinking) is predicted by score of 8 or more. | {AUDIT score} >=8 | *Babor, T. F., J. C. Higgins-Biddle, et al. (2001). "AUDIT: The alcohol use disorders identification test: Guidelines for use in primary health care."*  *Drummond, C., O. McBride, N. Fear and E. Fuller (2016). Alcohol dependence. Mental health and wellbeing in England: Adult Psychiatric Morbidity Survey. S. McManus, P. Bebbington, R. Jenkins and T. Brugha. Leeds, NHS Digital.* |
| nil | Alcohol | **Control: Hazardous / Harmful Alcohol Use.**  Uses inverse of the algorithmic diagnosis of hazardous drinking from AUDIT, excluding those who reported alcohol addiction in this questionnaire or reported at baseline they had stopped drinking due to illness, on drs advice or as a health precaution | AUDIT –ve ((Drinks alcohol 30414 = 0) OR **(**Typical drinks 20403 = “1 or 2” AND Six or more 20416 = “Never”))  AND  NOT {ever alcohol dependence}  AND  NOT reason for reducing amount of alcohol drunk 2664 = “ill health”, “doctor’s advice”or “health precaution” [1,2or3] | This is particularly strict control group to avoid including participants recovering from alcohol harm/dependence in the definition. |
| 3 | Addiction | **Case: Addiction ever.**  Endorses “Ever addicted to any substance or behaviour” | “Ever addicted to any substance or behaviour” 20401=1 |  |
| 1 | Addiction | **Case: Substance addiction.**  Endorses ever addicted to alcohol or drugs or medication. | Alcohol 20406 = Yes (1)  OR  Illicit/recreational drugs = Yes (1)  OR  Medication = Yes (1) |  |
| nil | Addiction | **Case: Current addiction:**  Endorses “addiction or dependence ongoing” | 20457=1 or 20504=1 or 20415=1 or 20432=1 |  |
| 1 | Addiction | **Case Alcohol dependence ever.**  Endorses “physically dependent on alcohol” | 20404=1 |  |
| nil | Addiction | **Control: Addiction ever.**  Not endorsing addiction, or other indicators of misuse: screening AUDIT in severe alcohol use disorder range or daily use of cannabis | NOT {ever addiction}  AND  NOT {AUDIT score >16}  AND  {daily cannabis} defined below |  |
| Exposures | | | | |
| S1, MD1 | Trauma | **Exposure: Childhood adverse events.**  Based on answers to the five questions of Childhood Trauma Screen (CTS), all scored 1-5. A score over the threshold on any question is screen positive. | [20489Felt loved as a child](http://biobank.ctsu.ox.ac.uk/crystal/field.cgi?id=20489) ≤3  OR  [20488Physically abused by family as a child](http://biobank.ctsu.ox.ac.uk/crystal/field.cgi?id=20488) ≥2  OR  [20487Felt hated by family member as a child](http://biobank.ctsu.ox.ac.uk/crystal/field.cgi?id=20487) ≥2  OR  [20490Sexually molested as a child](http://biobank.ctsu.ox.ac.uk/crystal/field.cgi?id=20490) ≥2  OR  [20491Someone to take to doctor when needed as a child](http://biobank.ctsu.ox.ac.uk/crystal/field.cgi?id=20491) ≤4 | CTS takes one question from each domain of the Childhood Trauma Questionnaire. Thresholds taken from thresholds for represented domain.  *Walker, E. A., et al. (1999). "Adult health status of women with histories of childhood abuse and neglect." The American Journal of Medicine* ***107****(4): 332-339* |
| S1, MD1 | Trauma | **Exposure: Adult adverse events.**  Based on answers to the five questions of Adult Trauma Screen (written for this questionnaire), all scored 1-5. A score over the threshold on any question is screen positive. | [20522Been in a confiding relationship as an adult](http://biobank.ctsu.ox.ac.uk/crystal/field.cgi?id=20522) ≤3  [20523Physical violence by partner or ex-partner as an adult](http://biobank.ctsu.ox.ac.uk/crystal/field.cgi?id=20523) ≥2  [20521Belittlement by partner or ex-partner as an adult](http://biobank.ctsu.ox.ac.uk/crystal/field.cgi?id=20521) ≥2  [20524Sexual interference by partner or ex-partner without consent as an adult](http://biobank.ctsu.ox.ac.uk/crystal/field.cgi?id=20524) ≥2  [20525Able to pay rent/mortgage](http://biobank.ctsu.ox.ac.uk/crystal/field.cgi?id=20525) ≤4 | Scoring algorithm based on Childhood Trauma Screen and consensus. Note some overlap with baseline questions included in loneliness score. |
| S1, MD1 | Trauma | **Exposure: Catastrophic trauma.**  Endorsed one or more events from checklist | [20531Victim of sexual assault](http://biobank.ctsu.ox.ac.uk/crystal/field.cgi?id=20531) = Yes, within last 12 months {2} OR Yes, but not in the last 12 months {1}  [20529Victim of physically violent crime](http://biobank.ctsu.ox.ac.uk/crystal/field.cgi?id=20529) = Yes, within last 12 months {2} OR Yes, but not in the last 12 months {1}  [20526Been in serious accident believed to be life-threatening](http://biobank.ctsu.ox.ac.uk/crystal/field.cgi?id=20526) = Yes, within last 12 months {2} OR Yes, but not in the last 12 months {1}  [20530Witnessed sudden violent death](http://biobank.ctsu.ox.ac.uk/crystal/field.cgi?id=20530) = Yes, within last 12 months {2} OR Yes, but not in the last 12 months {1}  [20528Diagnosed with life-threatening illness](http://biobank.ctsu.ox.ac.uk/crystal/field.cgi?id=20528) = Yes, within last 12 months {2} OR Yes, but not in the last 12 months {1}  [20527Been involved in combat or exposed to war-zone](http://biobank.ctsu.ox.ac.uk/crystal/field.cgi?id=20527) = Yes, within last 12 months {2} OR Yes, but not in the last 12 months {1} |  |
| nil | Cannabis | **Exposure: Cannabis ever.**  Endorsed taking cannabis at least once in life. | 20453 Ever taken cannabis >0 |  |
| S1, MD1 | Cannabis | **Exposure: Cannabis daily.**  Maximum frequency of taking cannabis when using is every day | 20454 frequency = every day {4} |  |
| nil | Cannabis | **Control: Cannabis ever.**  Reported no cannabis use | 20453 Ever taken cannabis = No {0} |  |
| Other | | | | |
| MD3 | Wellbeing | **Score: Wellbeing.**  Sum last three questions | General happiness 20458{scored 1-6}  + Happiness with health 20459{scored 1-6}  + Life meaningful 20460 {scored 1-5) |  |
| nil | Any | **Case: Any distress.**  Endorsing functional impairment or help-seeking due to mental distress, reports diagnosis or screens positive for specific condition | (Ever help for mental distress 20499 = yes)  OR  (Ever impairing mental distress = yes)  OR  (Mental health problem diagnosed 20544 = {1-18})  OR  Case {Depression ever, GAD ever, Addiction ever, Bipolar ever, Psychotic experiences, PTSD, Self harm ever} |  |
| nil | Any | **Control: Any distress.**  Not endorsing mental distress or conditions, and screens negative | Ever help for mental distress 20499 = no  AND  Ever impairing mental distress = yes  AND  NOT (Mental health problem diagnosed 20544 = {1-18 or -818 or -819})  AND  NOT Case {Depression ever, GAD ever, Addiction ever, Bipolar ever, Psychotic experiences, PTSD Self harm ever} | Inverse of case. Case plus control will contain all participants that had valid results in all sections |
| Non-MHQ | | | | |
| S1, MD1 | Other | **Exposure: Social isolation**  **Score > 1, where one mark each for:**  -“Including yourself, how many people are living together in your household? Include those who usually live in the house such as students living away from home during term time, partners in the armed forces or professions such as pilots”=0  -“How often do you visit friends or family or have them visit you?” = less than once a month  **-** “Which of the following [leisure/social activities] do you engage in once a week or more often?” =none | | Elovainio, M., C. Hakulinen, et al. "Contribution of risk factors to excess mortality in isolated and lonely individuals: an analysis of data from the UK Biobank cohort study." The Lancet Public Health **2**(6): e260-e266 |
| S1, MD1 | Other | **Exposure: Loneliness**  **Score > 1, where one mark each for:**  “Do you often feel lonely?” = yes  “How often are you able to confide in someone close to you?” = never or almost never | | Elovainio, M., C. Hakulinen, et al. "Contribution of risk factors to excess mortality in isolated and lonely individuals: an analysis of data from the UK Biobank cohort study." The Lancet Public Health **2**(6): e260-e266 |

.

# Appendix 3

STROBE Statement—Checklist of items that should be included in reports of ***cross-sectional studies***

|  | Item No | Recommendation | Davis et al MHQ paper |
| --- | --- | --- | --- |
| **Title and abstract** | 1 | (*a*) Indicate the study’s design with a commonly used term in the title or the abstract | Y |
|  |  | (*b*) Provide in the abstract an informative and balanced summary of what was done and what was found | Y |
| Introduction | | |  |
| Background/rationale | 2 | Explain the scientific background and rationale for the investigation being reported | Y |
| Objectives | 3 | State specific objectives, including any prespecified hypotheses | Y |
| Methods | | |  |
| Study design | 4 | Present key elements of study design early in the paper | Y |
| Setting | 5 | Describe the setting, locations, and relevant dates, including periods of recruitment, exposure, follow-up, and data collection | Referenced |
| Participants | 6 | (*a*) Give the eligibility criteria, and the sources and methods of selection of participants | Referenced |
| Variables | 7 | Clearly define all outcomes, exposures, predictors, potential confounders, and effect modifiers. Give diagnostic criteria, if applicable | Y - appendix |
| Data sources/ measurement | 8* | For each variable of interest, give sources of data and details of methods of assessment (measurement). Describe comparability of assessment methods if there is more than one group | Y |
| Bias | 9 | Describe any efforts to address potential sources of bias | N – not relevant |
| Study size | 10 | Explain how the study size was arrived at | N – not relevant |
| Quantitative variables | 11 | Explain how quantitative variables were handled in the analyses. If applicable, describe which groupings were chosen and why | Y – appendix |
| Statistical methods | 12 | (*a*) Describe all statistical methods, including those used to control for confounding | N – none used |
|  |  | (*b*) Describe any methods used to examine subgroups and interactions | N – none used |
|  |  | (*c*) Explain how missing data were addressed | N – not relevant |
|  |  | (*d*) If applicable, describe analytical methods taking account of sampling strategy | N – not used |
|  |  | (*e*) Describe any sensitivity analyses | N – not used |
| Results | | |  |
| Participants | 13* | (a) Report numbers of individuals at each stage of study—eg numbers potentially eligible, examined for eligibility, confirmed eligible, included in the study, completing follow-up, and analysed | Y |
|  |  | (b) Give reasons for non-participation at each stage | Y |
|  |  | (c) Consider use of a flow diagram | Y |
| Descriptive data | 14* | (a) Give characteristics of study participants (eg demographic, clinical, social) and information on exposures and potential confounders | Y |
|  |  | (b) Indicate number of participants with missing data for each variable of interest | Y – only in appendix |
| Outcome data | 15* | Report numbers of outcome events or summary measures | Y |
| Main results | 16 | (*a*) Give unadjusted estimates and, if applicable, confounder-adjusted estimates and their precision (eg, 95% confidence interval). Make clear which confounders were adjusted for and why they were included | Y – no precision given as sample size v large, and felt it would overstate the accuracy to use 95% CI or similar |
|  |  | (*b*) Report category boundaries when continuous variables were categorized | Y – in appendix |
|  |  | (*c*) If relevant, consider translating estimates of relative risk into absolute risk for a meaningful time period | N – not used |
| Other analyses | 17 | Report other analyses done—eg analyses of subgroups and interactions, and sensitivity analyses | N – not used |
| Discussion | | |  |
| Key results | 18 | Summarise key results with reference to study objectives | Y |
| Limitations | 19 | Discuss limitations of the study, taking into account sources of potential bias or imprecision. Discuss both direction and magnitude of any potential bias | Y – limitations of data given, less so limitations of analysis |
| Interpretation | 20 | Give a cautious overall interpretation of results considering objectives, limitations, multiplicity of analyses, results from similar studies, and other relevant evidence | Y |
| Generalisability | 21 | Discuss the generalisability (external validity) of the study results | Y |
| Other information | | |  |
| Funding | 22 | Give the source of funding and the role of the funders for the present study and, if applicable, for the original study on which the present article is based | Y |

*Give information separately for exposed and unexposed groups.

**Note:** An Explanation and Elaboration article discusses each checklist item and gives methodological background and published examples of transparent reporting. The STROBE checklist is best used in conjunction with this article (freely available on the Web sites of PLoS Medicine at http://www.plosmedicine.org/, Annals of Internal Medicine at http://www.annals.org/, and Epidemiology at http://www.epidem.com/). Information on the STROBE Initiative is available at www.strobe-statement.org.
